# The developmental transcriptome of the bamboo snout beetle *Cyrtotrachelus buqueti* and insights into candidate pheromone-binding proteins

**DOI:** 10.1371/journal.pone.0179807

**Published:** 2017-06-29

**Authors:** Hua Yang, Ting Su, Wei Yang, Chunping Yang, Lin Lu, Zhangming Chen

**Affiliations:** Key Laboratory of Ecological Forestry Engineering of Sichuan Province, College of Forestry, Sichuan Agricultural University, Chengdu, China; Institute of Plant Physiology and Ecology Shanghai Institutes for Biological Sciences, CHINA

## Abstract

*Cyrtotrachelus buqueti* is an extremely harmful bamboo borer, and the larvae of this pest attack clumping bamboo shoots. Pheromone-binding proteins (PBPs) play an important role in identifying insect sex pheromones, but the *C*. *buqueti* genome is not readily available for PBP analysis. Developmental transcriptomes of eggs, larvae from the first instar to the prepupal stage, pupae, and adults (females and males) from emergence to mating were built by RNA sequencing (RNA-Seq) in the present study to establish a sequence background of *C*. *buqueti* to help understand PBPs. Approximately 164.8 million clean reads were obtained and annotated into 108,854 transcripts. These were assembled into 24,338, 21,597, 24,798, 21,886, 24,642, and 83,115 unigenes for eggs, larvae, pupae, females, males, and the combined datasets, respectively. Unigenes were annotated against NCBI non-redundant protein sequences, NCBI non-redundant nucleotide sequences, Gene Ontology (GO), Protein family, Clusters of Orthologous Groups of Proteins/ Clusters of Eukaryotic Orthologous Groups (KOG), Swiss-Prot, and KEGG Orthology databases. A total of 17,213 unigenes were annotated into 55 sub-categories belonging to three main GO categories; 10,672 unigenes were classified into 26 functional categories by KOG classification, and 8,063 unigenes were classified into five functional KEGG categories. RSEM software for RNA sequencing showed that 4,816, 3,176, 3,661, 2,898, 4,316, 8,019, 7,273, 5,922, 5,844, and 4,570 genes were differentially expressed between larvae and males, larvae and eggs, larvae and pupae, larvae and females, males and females, males and eggs, males and pupae, females and eggs, females and pupae, and eggs and pupae, respectively. Of these, three were confirmed to be significantly differentially expressed between larvae, females, and males. Furthermore, PBP *Cbuq7577_g1* was highly expressed in the antenna of males. A comprehensive sequence resource of a desirable quality was constructed from developmental transcriptomes of *C*. *buqueti* eggs, larvae, pupae, and adults. This work enriches the genomic data of *C*. *buqueti*, and facilitates our understanding of its metamorphosis, development, and response to environmental change. The identified candidate PBP *Cbuq7577_g1* might play a crucial role in identifying sex pheromones, and could be used as a targeted gene to control *C*. *buqueti* numbers by disrupting sex pheromone communication.

## Introduction

The bamboo snout beetle *Cyrtotrachelus buqueti* Guerin-Meneville (Coleoptera: Curculionidae) is widely distributed in China, Vietnam, Burma, Thailand, and other Southeast Asian countries [1, 2]. *C*. *buqueti* mainly attacks the shoots of 28 bamboo species, including *Bambusa*, *Dendrocalamopsis*, and *Dendrocalamus*, while the larvae bore into the shoots of clumping bamboo species such as *Phyllostachys pubescens*, *Neosinocalamus affinis*, *Bambusa*. *textiles*, *Bambusa*. *pervariabilis*, and *Bambusa*. *oldhamii* [3].

In Sichuan Province, China, nearly 67,000 hm^2^ of forests are affected by *C*. *buqueti* every year. The damage rate is typically 50%–80%, although in severe cases this may reach 100%. *C*. *buqueti* is therefore a major forest pest, and the severity of the damage caused has become an important factor restricting the development of bamboo for paper making [4]. In April 2003, the State Forestry Administration of the People’s Republic of China released a list of 156 harmful forest organisms, which included *C*. *buqueti* among other pests and harmful mites.

Current studies of *C*. *buqueti* are mainly focused on its biological characteristics and chemical control methods. Wang et al. [4] conducted a preliminary study on its reproductive behavior, while Chen et al. [5] analyzed the harmful activity and pest control of *C*. *buqueti*. Yang et al. [3] studied the relationship between *C*. *buqueti* larval density and wormhole number and bamboo shoot damage. A later study [6] investigated the behavioral and electroantennogram (EAG) responses of *C*. *buqueti* adults to host volatiles and their body extracts, revealing that pheromones released by both male and female *C*. *buqueti* strongly attract members of the opposite sex, and that the addition of host plants can strengthen the attraction between sexes. On this basis, the main components of bamboo shoot volatiles and EAG responses of *C*. *buqueti* to bamboo shoot volatiles were examined [7], and Mang et al. [1] extracted and identified the cuticular semiochemical components of *C*. *buquti* adults.

Insect odorant binding proteins (OBPs) are mainly divided into PBPs, general OBPs (GOBPs), and antennae-binding proteins. The main function of PBPs is to bind and transport sex pheromones, while GOBPs may be associated with the general binding of biogenic volatile organic compounds [8]. Because PBPs selectively recognize sex pheromone components of very similar structures [9], they play an important role in the exchange of information between male and female insects and in reproductive isolation.

Researchers have identified several PBPs in a single species that are encoded by different genes [10, 11]. PBP genes have been cloned from *Sesamia nonagrioides* [12], *Bombyx mori* [13], *Spodoptera litura* [14], *Spodoptera exigua* [15], *Antherea polyphemus* [16], *Leucophea maderea* [17], *Drosophila melanogaster* [18], and *Apis mellifera* [19], including PBP1, PBP2, and PBP3. The homology of the encoded amino acid sequences is between 32% and 92% [20]. The identification of functional olfactory molecules will also facilitate the development of attractants for baits in management systems.

In the present study, we used RNA sequencing (RNA-seq) to identify developmental stage-specific genes by building transcriptomes of eggs, larvae from the first instar to the prepupal stage, pupae, and adults (females and males) from emergence to mating (3 days old). We identified differentially expressed genes among eggs, larvae, pupae, females, and males by comparative transcriptome analysis. We also screened *C*. *buqueti* candidate PBPs because the olfactory system is crucial to sexual communication and reproductive isolation in insects. Finally, differentially expressed candidate PBPs underwent transcriptome data validation.

## Material and methods

### Insect rearing and collection

Eggs, larvae, and pupae of *C*. *buqueti* were collected in July 2015 from in the bamboo planting base of Lushan City, Sichuan Province, China (102°91′N,30°13′E). The field studies did not involve endangered or protected species, and no specific permission was required for the research activity at this location. Pupae were reared in our laboratory at 25°C ± 1°C and 70 ± 10% relative humidity, with a 12L: 12D photoperiod. Adults were used in the experiment 3 days after emergence [21]. Female and male adults were placed on ice and were quickly dissected into the head (without antenna), thorax (without thoracic legs), abdomen, antenna, and thoracic legs for qRT-PCR analysis. All samples were immediately frozen in liquid nitrogen and stored at −80°C until use. Each sample contained eggs, larvae, pupae, males, females and adult tissues from at least five insects, respectively. After mixed sample, three biological replicates were conducted for each treatment.

### RNA extraction and sequencing

Eggs, mixed larvae from the first instar to the prepupal stage, pupae, and adults (females and males) from emergence to mating (3 days old) were prepared for RNA extraction. Total RNA was extracted using TRIzol reagent (Qiagen, China Shanghai). The concentration of total RNA was quantified using a spectrophotometer (Implen, Westlake Village, CA), and the RNA integrity was tested using 1.5% agarose gel electrophoresis. After RNA extraction, mRNAs were purified using a Poly A T tract mRNA isolation system and collected using an RNeasy RNA reagent. Mixed mRNAs were fragmented into 300–800 bp pieces using RNA Fragment reagent (Illumina), and the pieces were collected using an RNeasy RNA cleaning kit (Qiagen). Subsequently, RNA fragments were copied into first strand cDNA using MMLV reverse transcriptase (TaKaRa, Dalian Liaoning, Chinese) and random primers. Second strand cDNA synthesis was performed using DNA Polymerase I and RNase H. The Illumina HiSeq2000 system and 100 paired-end reads were used for sequencing. Clean reads were obtained by removing adaptors, low-quality reads, and contaminated reads from raw sequence reads. Statistical analysis of the sequence length was performed to ensure sequence purity.

### Assembly and functional annotation

Raw sequence data reads in fasta format were firstly processed through in-house Perl scripts [22]. In this step, clean data (clean reads) were obtained by removing reads containing adapter, ploy-N and low-quality reads form raw read data [23, 24]. At the same time, Q20, Q30, GC-content, and sequence duplication level of the clean data were calculated [24]. All downstream analyses were based on clean high-quality data.

A flow chart of transcriptome assembly as described by Grabherret et al. [25] was used in the present analyses. A Perl pipeline as described by Haas et al. [22] was used for analyzing sequence data. As suggested by Haas et al.[22], if multiple sequencing runs are conducted for a single experiment, these reads may be concatenated into two files in the case of paired-end sequencing. The left files (read 1 files) from all samples were pooled into a single large left.fq file, and right files (read 2 files) into a single large right.fq file. Transcriptome assembly was accomplished based on the left.fq and right.fq using Trinity (http://trinityrnaseq.github.io) with min_kmer_cov set to two by default and all other parameters set default. The assembled unigenes were annotated by BLASTX and ESTScan against Nr, Nt, Pfam, KOG/COG, Swiss-Prot, KO, and GO databases (E < 10^−5^), and the best annotations were selected [23, 24, 26] (S1 Table). Differentially expressed genes were selected by log2 fold change > 1 and q value < 0.005 according the method of DESeq[27]. The nucleotide sequences of each identified *PBP* gene are listed in S2 Table.

### Homology analysis

A neighbor-joining (NJ) tree was constructed with MEGA version 5.0 [28] and the Jones-Taylor-Thornton model. The olfactory gene sequences of other coleopteran insects were first transcribed into their amino acid sequences using the ORF finder (http://www.ncbi.nlm.nih.gov/gorf/gorf.html). Olfactory genes of other coleopteran species were obtained from the NCBI databases. Bootstrap support values were based on 1000 replicates. All of the candidate olfactory genes were named according to the nomenclature system described previously [29, 30].

### RT-PCR and qRT-PCR validation

Specific primer pairs were derived from the transcriptome data, and primer pairs for each gene were designed to amplify 100–200 bp products, which were verified by sequencing. A conventional RT-PCR (Bio-Rad S1000, US) analysis was performed for each primer pair using r*Taq* DNA polymerase (TaKaRa, Dalian, Liaoning, China) before the qRT-PCR (Bio-Rad CFX96, US) analysis to ensure that the correct products were amplified and no primer dimers were present. The qRT-PCR analysis was carried out using an Mx 3000P detection system (Stratagene, La Jolla, CA) as described previously, with thermal cycler parameters of 1 min at 95°C, then 40 cycles of 30 s at 95°C, 30 s at 60°C, and 30 s at 72°C. *GAPDH* of *C*. *buqueti* (GenBank accession number: SAMN06176790) was used as the housekeeping gene. A standard curve was derived from 10-fold serial dilutions of plasmid containing the target DNA segment to determine the PCR efficiency and for quantifying the amount of target mRNA. All primers tested gave amplification efficiencies of 90%–100%. For each treatment, three biological replicates were conducted. qRT-PCR data were analyzed by the 2^-ΔΔCT^ method[31]. The primers used in this experiment were designed with Primer premier 5.0 and Oligo 6.0 and are listed in S3 Table. The qRT-PCR data were analyzed and output as PDF files using Graphpad 5.0.

## Results

### Illumina sequencing and assembly

Clean reads were obtained from raw reads after the removal of those reads with low quality, adapters, duplicated, and ambiguous. This resulted in a total of 31,469,916, 36,773,825, 32,128,345, 33,070,448, and 31,434,121 clean reads in eggs, larvae, pupae, females, and males of *C*. *buqueti*, respectively. All clean reads were assembled into transcripts by Trinity software; the longest copy of redundant transcripts was regarded as a unigene [22, 24, 25]. A total of 108,854 transcripts were obtained and assembled into 83,115 unigenes. Many unigenes were 200–1000 bp in length (Table 1), while approximately 14.7% unigenes exceeded 1000 bp, and 7.2% exceeded 2000 bp (Table 1).

**Table 1:** Number and length of transcripts and unigenes.

	Egg	Larval	Pupae	Female	Male
Raw reads	31,712,272	37,074,123	32,428,131	33,314,198	31,668,491
Clean reads	31,469,916	36,773,825	32,128,345	33,070,448	31,434,121
Clean bases	3.93G	4.60G	4.02G	4.13G	3.93G
Q20%	97.41	97.31	97.27	97.45	97.38
Q30%	94.06	93.88	93.81	94.13	94.01
GC%	38.79	39.37	42.27	38.37	38.81
	Transcripts		Unigenes		
200–500 bp	66,112		57,464		
500–1 k bp	18,103		13,417		
1 k-2 k bp	10,352		6,238		
>2 k bp	14,287		5,996		
Total number	108,854		83,115		
Min length	201		201		
Mean length	1,012		710		
Max length	27,826		27,826		
N50	2,638		1341		
N90	322		260		
Total nucleotides	110,129,824		59,040,873		
	Number of Unigenes		Percentage%		
Annotated in NR	21,058		25.33		
Annotated in NT	5,890		7.08		
Annotated in KO	8,063		9.70		
Annotated in SwissProt	14,748		17.74		
Annotated in PFAM	17,105		20.57		
Annotated in GO	17,213		20.70		
Annotated in KOG	10,672		12.84		
Annotated in all databases	2,795		3.36		
Annotated in at least one databases	27,017		32.50		

### Annotation of unigenes

The assembled unigenes were annotated against NCBI non-redundant protein sequences (Nr), NCBI non-redundant nucleotide sequences (Nt), KEGG Orthology (KO), Swiss-Prot, Protein family (Pfam), Gene Ontology (GO), and Clusters of Eukaryotic Orthologous Groups/Clusters of Orthologous Groups of Proteins (KOG/COG) databases. A total of 24,798 unigenes were annotated in *C*. *buqueti* pupae (CP), 24,338 in eggs (CE), 21,597 in larval (CP), 24.642 in male (CM), 21,886 in female (CF), 1,387 in CP-specific, 1,296 in CE-specific, 801 in CL-specific, 1,051 in CM-specific, 735 in CF-specific, 8,989 in Common, 83,115 in CP-CE-CL-CF-CM Combined datasets. (Table 2). The number and percentage of unigenes annotated in these databases were counted. The Nr database had the best match against the CP-CE-CL-CF-CM Combined dataset (21,058, 25.33%) (Table 2), while Swiss-Prot (14,748, 17.74%), Pfam (17,105, 20.57%), and GO (17,213, 20.70%) shared similar quantities (Table 2) (S1–S15 Texts).

**Table 2:** Unigenes annotated in different databases.

	CP		CE		CL		CM		CF		CP-specific	
	NO.	PCT(%)	NO.	PCT(%)	NO.	PCT(%)	NO.	PCT(%)	NO.	PCT(%)	NO.	PCT(%)
NR	11792	47.55%	11812	48.53%	11099	51.39%	12273	49.80%	11439	52.27%	125	9.01%
NT	3667	14.79%	3707	15.23%	3440	15.93%	3726	15.12%	3598	16.44%	27	1.95%
KO	5228	21.08%	5183	21.30%	5036	23.32%	5381	21.84%	5202	23.77%	34	2.43%
Swissprot	9091	36.66%	9065	37.24%	8697	40.27%	9352	37.95%	8896	40.65%	56	4.04%
PFAM	9806	39.54%	9770	40.14%	9269	42.92%	10046	40.77%	9503	43.42%	120	8.63%
GO	7524	30.34%	7521	30.90%	7168	33.19%	7770	31.53%	7354	33.60%	89	6.42%
KOG	7049	28.43%	7013	28.82%	6747	31.24%	7236	29.36%	6941	31.71%	43	3.10%
Total NO.	24798		24338		21597		24642		21886		1387	
	CE-specific		CL-specific		CM-specific		CF-specific		Common		CP-CE-CL-CM-CF combined	
	NO.	PCT(%)	NO.	PCT(%)	NO.	PCT(%)	NO.	PCT(%)	NO.	PCT(%)	NO.	PCT(%)
NR	134	10.37%	93	11.57%	139	13.23%	91	12.38%	7578	84.30%	21058	25.33
NT	104	8.05%	20	2.50%	34	3.20%	23	3.13%	2693	29.96%	5890	7.08
KO	41	3.19%	36	4.45%	50	4.76%	39	5.26%	3921	43.62%	8063	9.7
Swissprot	103	7.97%	70	8.70%	90	8.53%	66	8.98%	6438	71.62%	14748	17.74
PFAM	120	9.23%	93	11.65%	112	10.69%	82	11.11%	6436	71.60%	17105	20.57
GO	87	6.69%	71	8.91%	81	7.74%	62	8.48%	5070	56.40%	17213	20.7
KOG	55	4.22%	48	5.99%	53	5.04%	38	5.22%	5200	57.85%	10672	12.84
Total NO.	1296		801		1051		735		8989		83115	

CP: Unigenes of *Cyrtotrachelus buqueti* pupae; CE: Unigenes of *C*. *buqueti* eggs; CL: Unigenes of *C*. *buqueti* larvae; CM: Unigenes of *C*. *buqueti* males; CF: Unigenes of *C*. *buqueti* females; CP-specific: Specific unigenes of *C*. *buqueti* pupae; CE-specific: Specific unigenes of *C*. *buqueti* eggs; CL-specific: Specific unigenes of *C*. *buqueti* larvae; CM-specific: Specific unigenes of *C*. *buqueti* males; CF-specific: Specific unigenes of *C*. *buqueti* females; Common: Common unigenes of *C*. *buqueti* pupae, eggs, larvae, males, and females; CP-CE-CL-CM-CF Combined: Total unigenes of *C*. *buqueti* pupae, eggs, larvae, males, and females. NO: number; PCT (%): percentage (%); NR: NCBI non-redundant protein sequences; NT: NCBI non-redundant nucleotide sequences; KO: KEGG Orthology; Swissprot: A manually annotated and reviewed protein sequence database; PFAM: Protein family; GO Gene Ontology; KOG: Clusters of Orthologous Groups of protein; Total NO: Total number of annotated unigenes.

**Fig 1:**
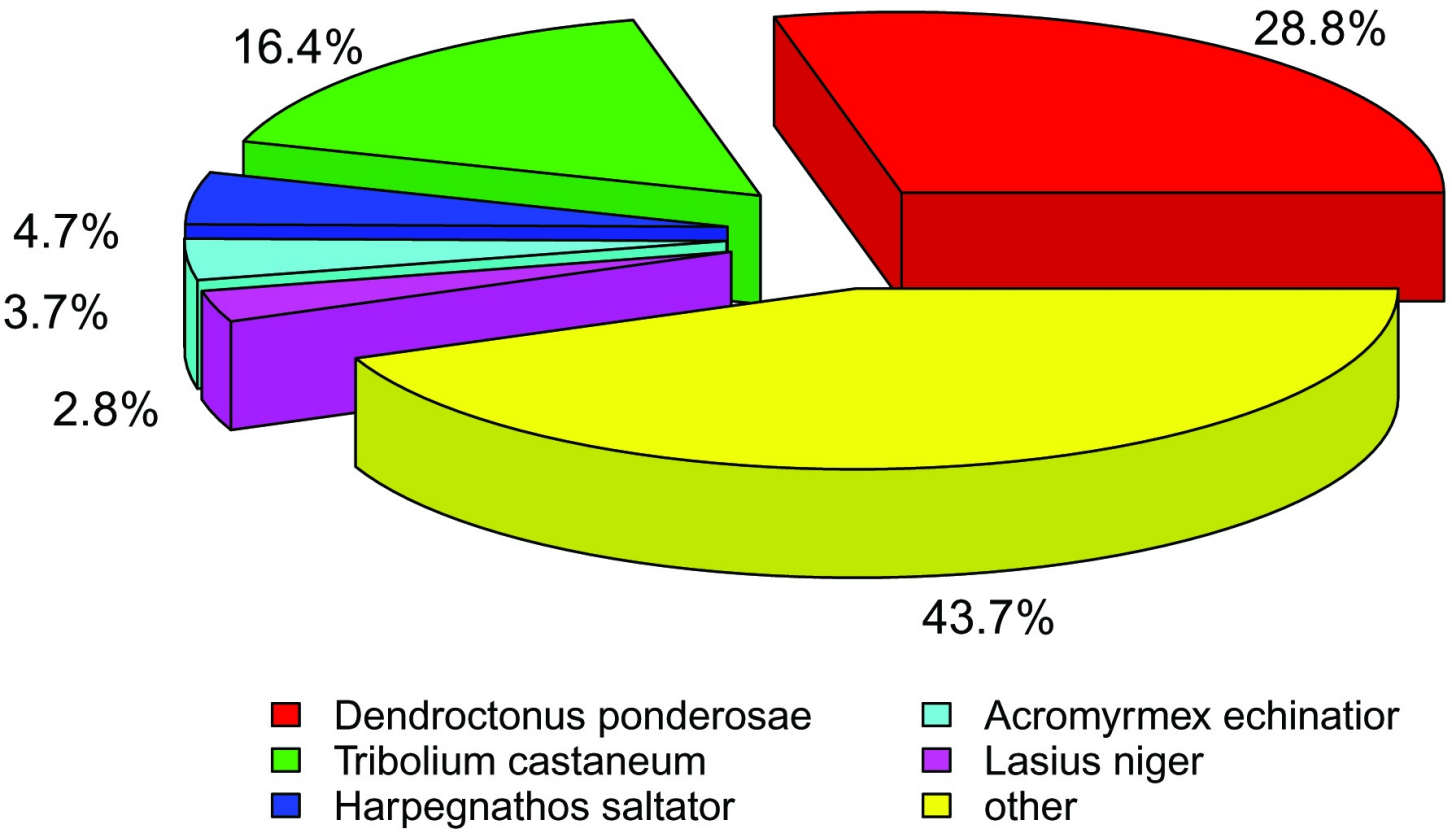
Percentage numbers of the five most abundant annotated species.

After functional annotation, the numbers of sequences from different species that matched the bamboo snout beetle unigenes were calculated from the annotation characteristics. As displayed in Fig 1, the five species were *Dendroctonus ponderosae* (28.8%), *Tribolium castaneum* (16.4%), *Harpegnathos saltator* (4.7%), *Acromyrmex echinatior* (3.7%), and *Lasius niger* (2.8%), representing around 56% of all the species that were annotated.

### Functional annotation results

**Fig 2:**
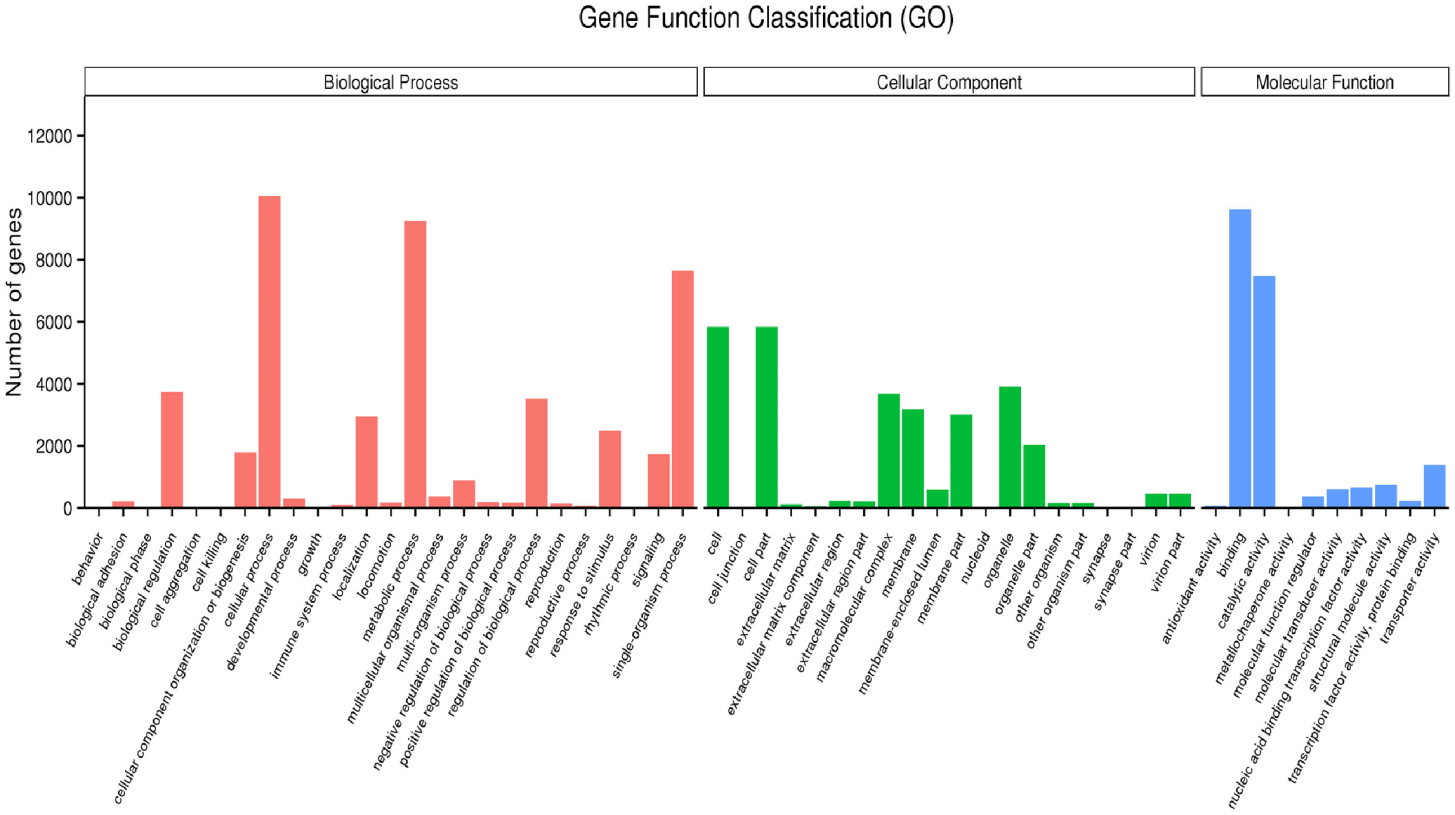
Histogram of GO classification of unigenes.

A total of 17,213 unigenes were annotated into 55 sub-categories belonging to three main GO categories: biological process (BP), cellular component (CC), and molecular function (MF) (Fig 2). There were 25 sub-categories in BP, 20 in CC, and 10 in MF. The top 10 sub-categories were cellular process (10,055 unigenes), metabolic process (9,255 unigenes), single organism process (7,651 unigenes), biological regulation (3,728 unigenes), cell (5,827 unigenes), cell part (5,827 unigenes), organelle (3,914 unigenes), macromolecular complex (3,682 unigenes), binding (9,624 unigenes), and catalytic activity (7,481 unigenes) (S16 Text).

**Fig 3:**
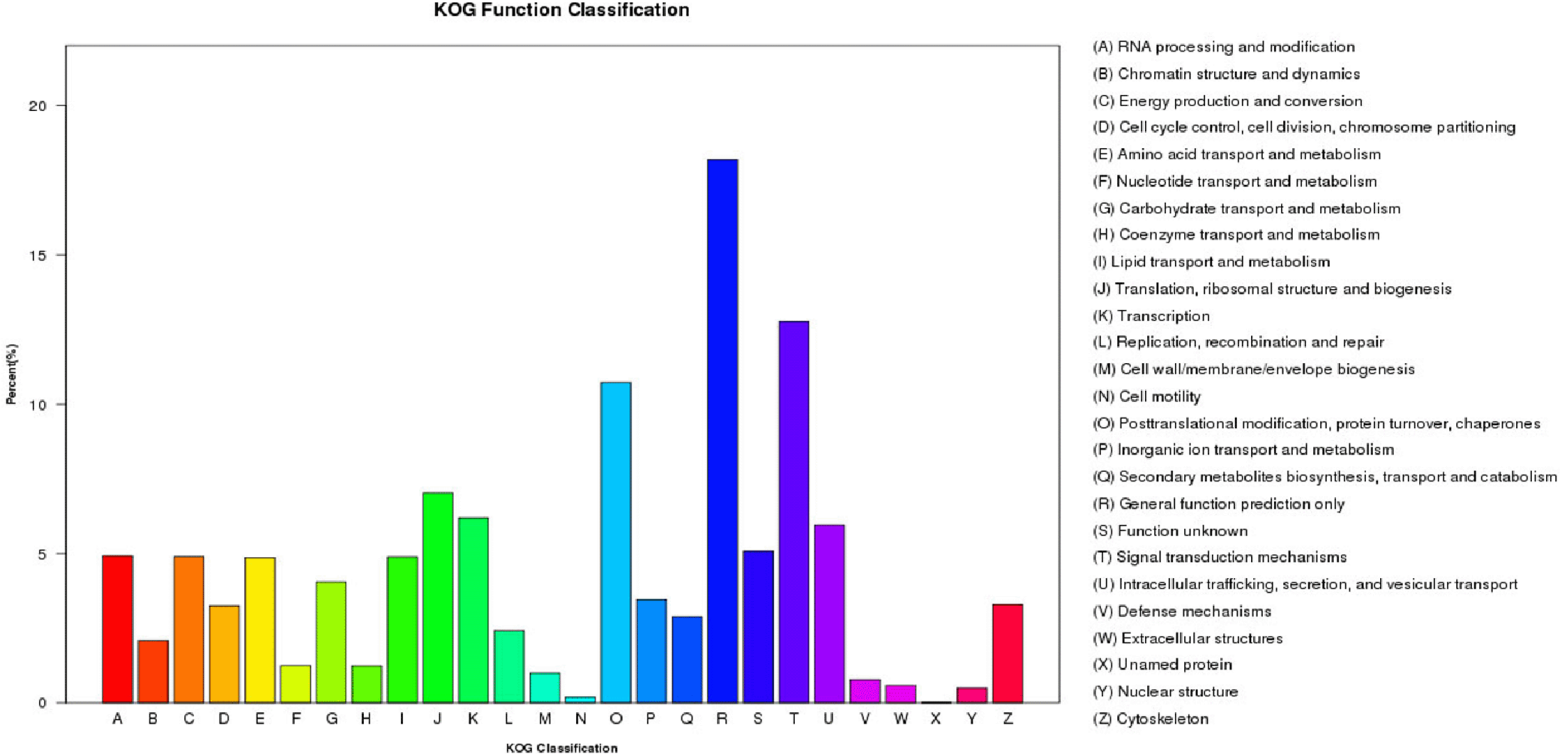
Histogram of KOG classification of unigenes.

KOG classification placed 10,672 unigenes into 26 functional categories (Fig 3). The cluster of ‘general function prediction only’ was the largest group (1,940 unigenes), followed by ‘signal transduction mechanisms’ (1,362 unigenes), and ‘posttranslational modification, protein turnover, chaperons’ (1,144 unigenes). The top three categories had 41.7% of unigenes annotated to the KOG database (S17 Text).

**Fig 4:**
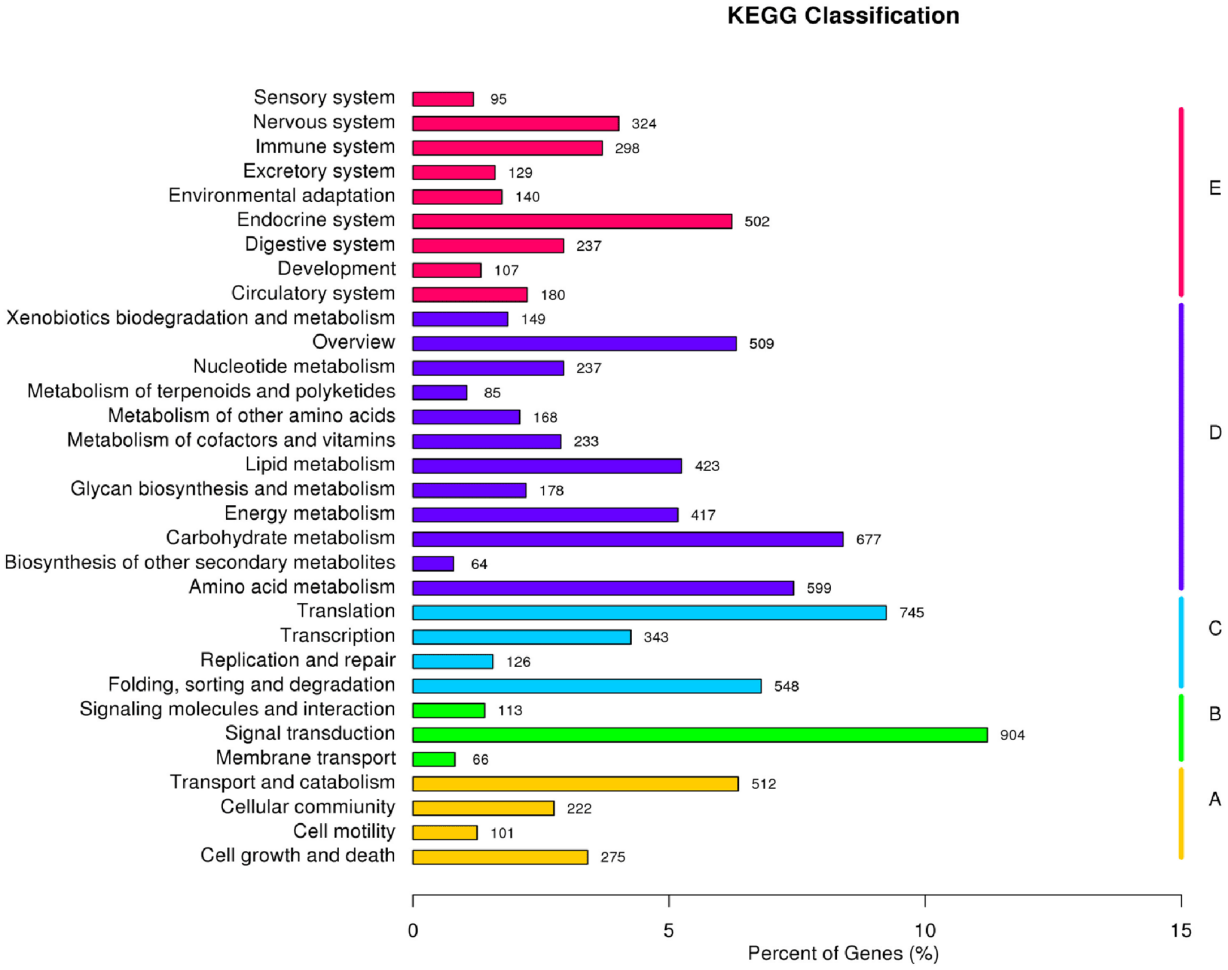
Histogram of KEGG classification of unigenes. **a** Cellular Processes. **b** Environmental Information Processing. **c** Genetic Information Processing. **d** Metabolism. **e** Organismal Systems.

In total, 8,063 unigenes were classified into five KEGG functional categories (Fig 4): cellular process (1,110 unigenes, 11.44% of which were annotated to the KEGG database), environmental information processing (1,083, 11.16%), genetic information processing (1,762, 18.15%), metabolism (3,739, 38.52%), and organismal system (2,012, 20.73%) (S18 Text). The top three subcategories out of a total of 32 were ‘signal transduction’, ‘translation’, and ‘carbohydrate metabolism’.

### CDS prediction

**Fig 5:**
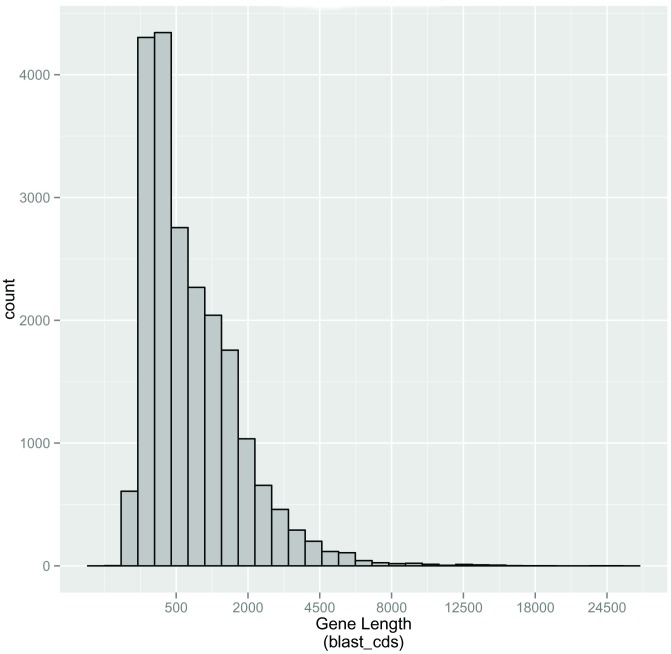
Length distribution of unigenes predicted protein coding sequence (CDS) using BLAST.

**Fig 6:**
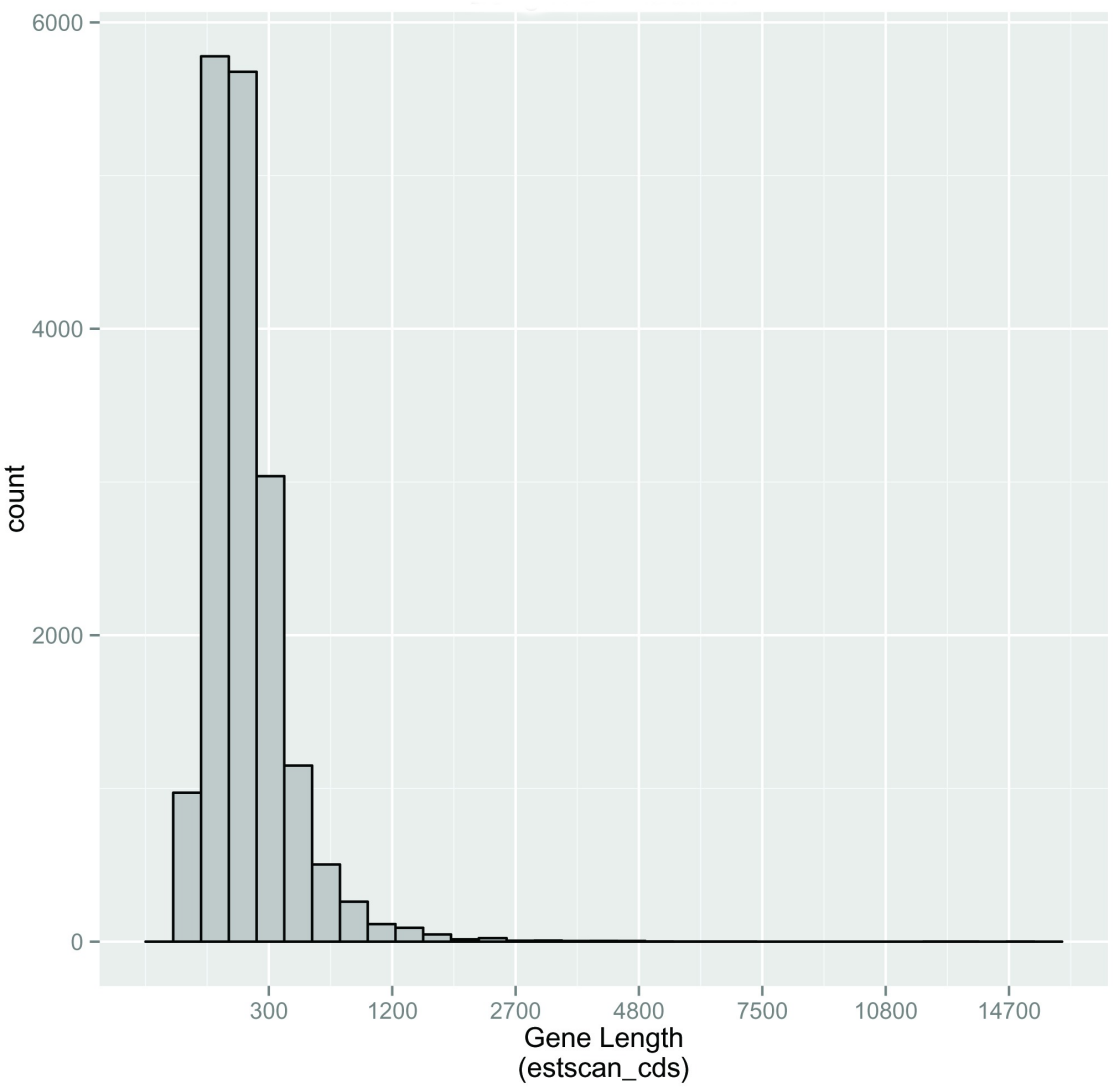
Length distribution of assembled unigenes predicted protein coding sequence (CDS) using ESTScan.

A total of 21,102 (25.39%) unigenes were predicted via BLASTX with an E-value threshold of 10^−5^ in the Nr, and the Swiss-Prot database (Figs 5 and 6). Among these, 14,998 unigenes were in the length of more than 300 bp (Fig 5). Furthermore, 17,703 (21.30%) unigenes were then predicted using ESTScan, which identified 3,415 unigenes of more than 300 bp in length (Fig 6).

### Differentially expressed genes

**Fig 7:**
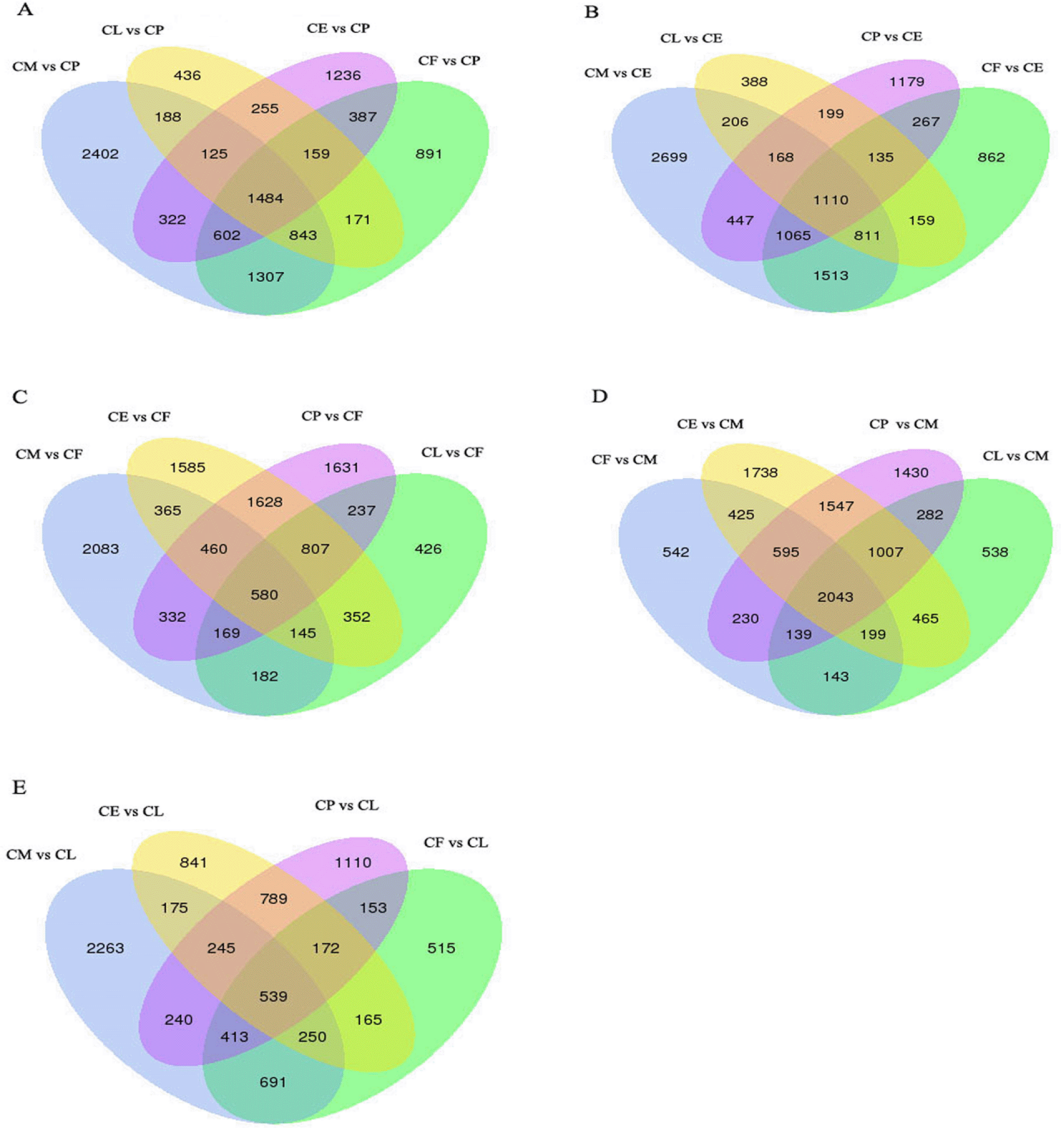
Venn diagram of the number of differentially expressed genes in CE, CL, CP, CM, and CF. CE: *C*. *buqueti* eggs, CL: *C*. *buqueti* larvae, CP: *C*. *buqueti* pupae, CM: *C*. *buqueti* males, CF: *C*. *buqueti* females.

A total of 7,273, 3,661, 4,570, and 5,844 genes were differentially expressed between pupae and males, pupae and larvae, pupae and eggs, and pupae and females, respectively, with 1,484 common to pupae, males, larvae, eggs, and females (Fig 7A). A total of 8,019, 3,176, 4,570, and 5,922 genes were differentially expressed between eggs and males, eggs and larvae, eggs and pupae, and eggs and females, respectively, with 1,110 common to eggs, males, females, pupae, and larvae (Fig 7B). A total of 4,316, 5,922, 5,844, and 2,898 genes were differentially expressed between females and males, females and eggs, females and pupae, and females and larvae, respectively, with 580 common to females, males, eggs, pupae, and larvae (Fig 7C). A total of 4,316, 8,019, 7,273, and 4,816 genes were differentially expressed between males and females, males and eggs, males and pupae, and males and larvae, respectively, with 2,043 common to males, females, eggs, pupae, and larvae (Fig 7D). A total of 4,816, 3,176, 3,661, and 2,898 genes were differentially expressed between larvae and males, larvae and eggs, larvae and pupae, and larvae and females, respectively, with 539 common to larvae, males, eggs, pupae, and females (Fig 7E) (S19–S28 Texts).

**Fig 8:**
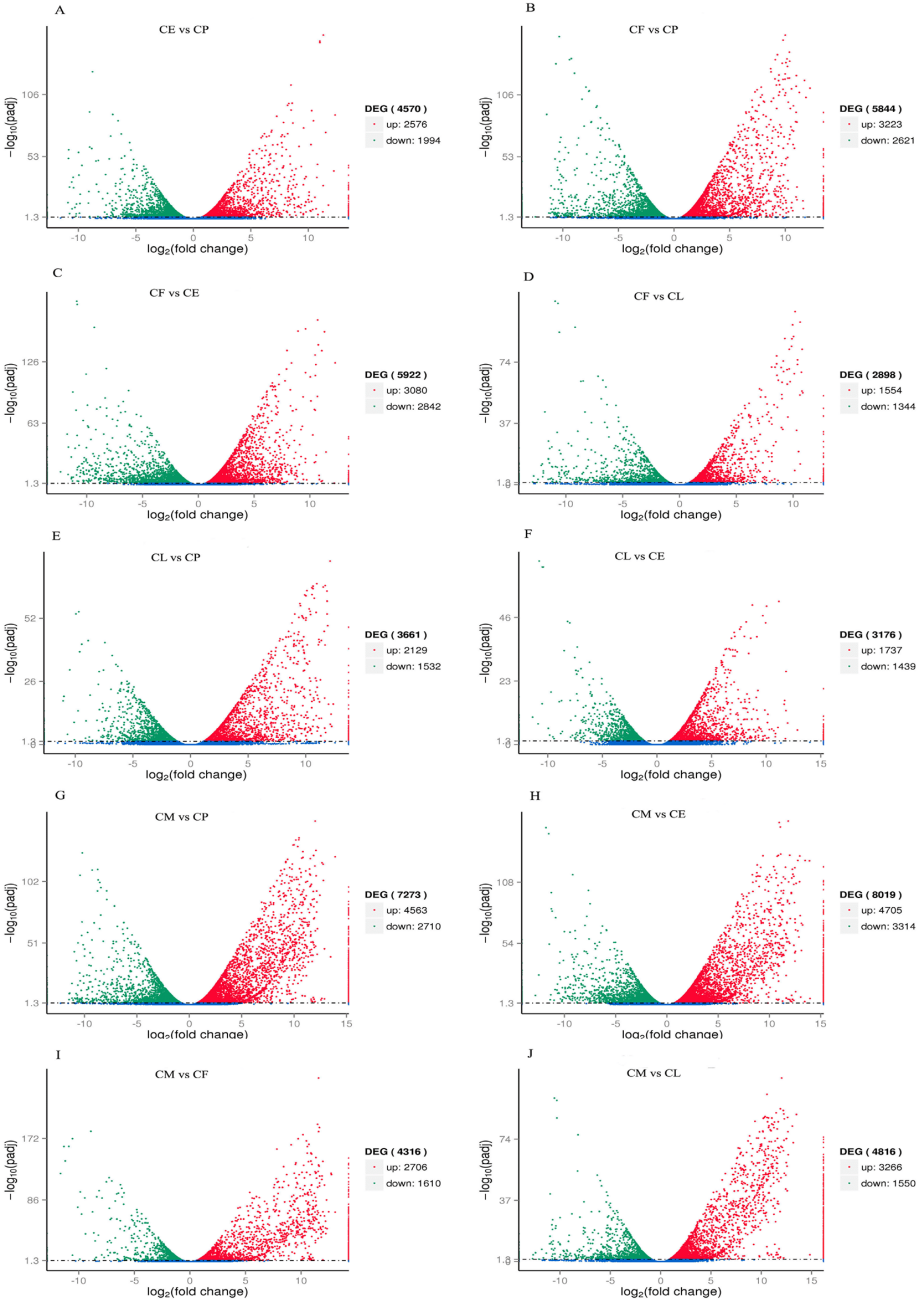
Volcano plot of differentially expressed genes in eggs, larvae, pupae, males, and females. **a** Volcano plot of differentially expressed genes between CE and CP. **b** Volcano plot of differentially expressed genes between CF and CP. **c** Volcano plot of differentially expressed genes between CF and CE. **d** Volcano plot of differentially expressed genes between CF and CL. **e** Volcano plot of differentially expressed genes between CL and CP. **f** Volcano plot of differentially expressed genes between CL and CE. **g** Volcano plot of differentially expressed genes between CM and CP. **h** Volcano plot of differentially expressed genes between CM and CE. **i** Volcano plot of differentially expressed genes between CM and CF. **j** Volcano plot of differentially expressed genes between CM and CL. Splashes represent different genes. Blue splashes means genes without significant different expression. Red splashes mean significantly upregulated genes. Green splashes mean significantly downregulated genes. CE, CL, CP, CM, and CF represent eggs, larvae, pupae, males, and females of *C*. *buqueti*, respectively.

More genes were shown to be expressed in eggs than in pupae, in females than in pupae, in females than in eggs, in females than in larvae, in larvae than in pupae, in larvae than in eggs, in males than in pupae, in males than in eggs, in males than in females, and in males than in larvae (2,576, 3,223, 3,080, 1,554, 2,129, 1,737, 4,563, 4,705, 2,706, and 3,266, respectively; Fig 8). Conversely, fewer genes were shown to be expressed in eggs than in pupae, in females than in pupae, in females than in eggs, in females than in larvae, in larvae than in pupae, in larvae than in eggs, in males than in pupae, in males than in eggs, in males than in females, and in males than in larvae (1,994, 2,621, 2,842, 1,344, 1,532, 1,439, 2,710, 3,314, 1,610, and 1,550, respectively; Fig 8).

### Phylogenetic analysis of candidate PBP

**Fig 9:**
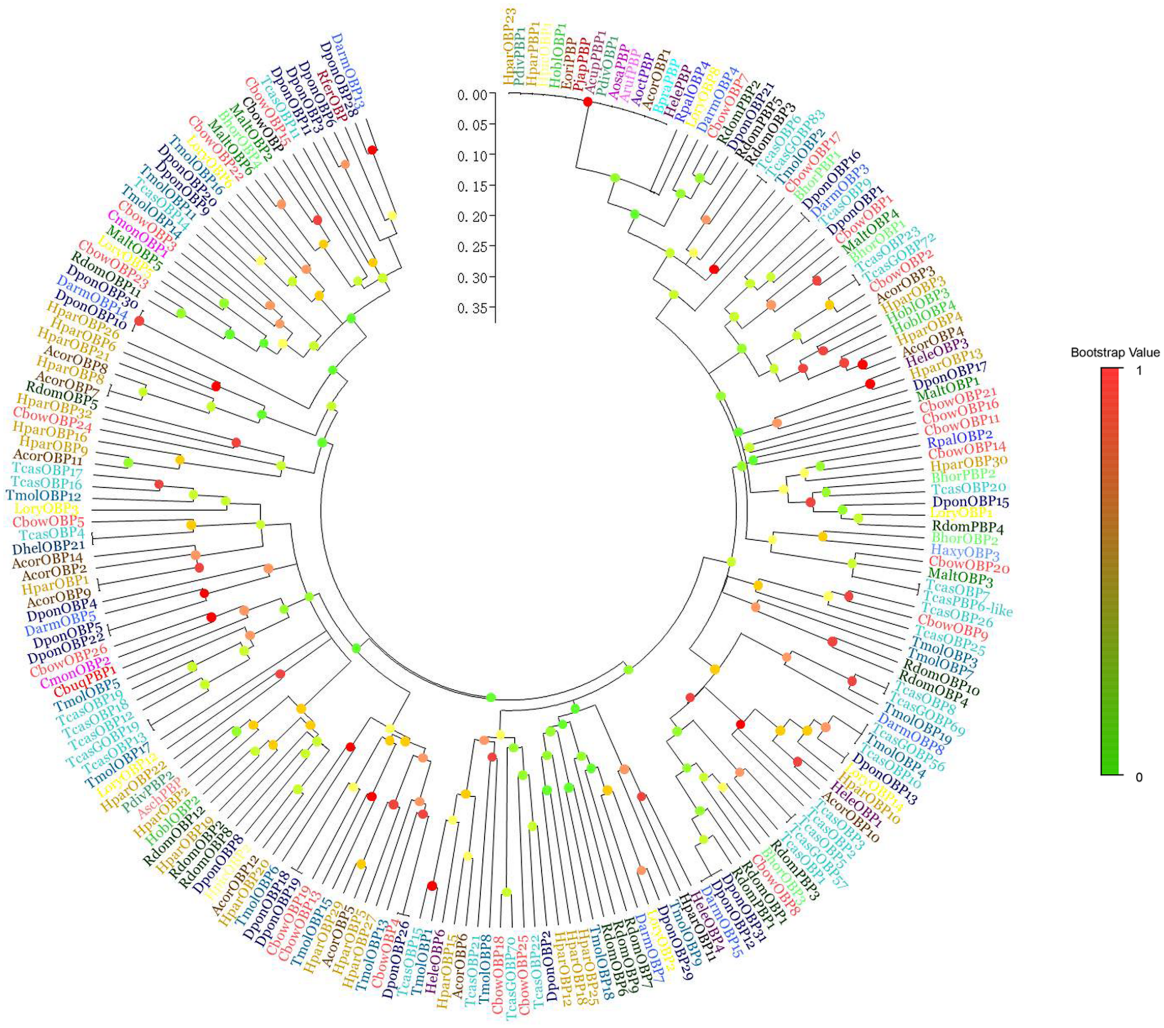
Neighbor-joining tree of CbuqPBP1. Values indicated at the nodes are bootstrap values based on 1000 replicates; scale bar = 0.1. Cbuq: *Cyrtotrachelus buqueti*; Rdom: *Rhyzopertha dominica*; Dhel: *Dastarcus helophoroides*; Dpon: *Dendroctonus ponderosae*; Hele: *Hylamorpha elegans*; Rfer: *Rhynchophorus ferrugineus*; Acor: *Anomala corpulenta*; Malt: *Monochamus alternatus*; Tmol: *Tenebrio molitor*; Rpal: *Rhynchophorus palmarum*; Aosa: *Anomala osakana*; Pjap: *Popillia japonica*; Hpar: *Holotrichia parallela*; Hobl: *Holotrichia oblita*; Tcas: *Tribolium castaneum*; Darm: *Dendroctonus armandi*; Cmon: *Cryptolaemus montrouzieri*; Cbow: *Colaphellus bowringi*; Lory: *Lissorhoptrus oryzophilus*; Bhor: *Batocera horsfieldi*; Bpra: *Brachysternus prasinus*; Haxy: *Harmonia axyridis*; Aruf: *Anomala rufocuprea*; Asch: *Anomala schonfeldti*; Hpic: *Heptophylla picea*; Pdiv: *Phyllopertha diversa*; Aoct: *Anomala octiescostata*; Acup: *Anomala cuprea*; Eori: *Exomala orientalis*. The olfactory genes from different species were marked with different colors (S32 Text).

We constructed a phylogenetic tree comparing *Cbuq7577_g1* (Gene name: *CbuqPBP1*) and the olfactory genes from 28 coleopteran insects (Fig 9) (S29 Text). In this tree, the olfactory genes are well separated with strong bootstrap support. *Cryptolaemus montrouzieri CmonOBP2* and *Colaphellus bowringi CbowOBP26*, along with *CbuqPBP1*, were located on the same clade.

### Expression profiles of pheromone-binding proteins

We identified 19 candidate *PBPs* through the Nr database (nucleotide sequences are listed in S30 Text). Of these, significant differences in expression profiles were identified in 13 candidate *PBPs* in male adults and larvae (Table 3), 10 candidate *PBPs* in female adults and larvae (Table 4), and three candidate *PBPs* in female and male adults (Table 5).

**Table 3:** Differentially expressed PBPs between males and larvae.

Gene	Readcount-Male	Readcount-Larvae	log_2_Fold-change	q
*Cbuq 12614_g1*	1,145.1918	4,063.5763	–1.8272	<0.005*
*Cbuq 15552_g1*	118.3438	261.6222	–1.1445	>0.005
*Cbuq 16395_g1*	729.5298	9,356.5343	–3.6809	<0.005*
*Cbuq 25979_g1*	1,331.5847	3,409.7511	–1.3565	<0.005*
*Cbuq 29237_g1*	12.5272	218.5220	–4.1246	<0.005*
*Cbuq 37516_g1*	7,153.6209	30.8767	7.8560	<0.005*
*Cbuq 46750_g1*	631.9583	414.4649	0.6086	>0.005
*Cbuq 47175_g1*	16,357.2963	37,877.4378	–1.2114	>0.005
*Cbuq 61968_g1*	1,539.7646	932.1776	0.7240	>0.005
*Cbuq 61968_g2*	1,012.476	4,448.6403	–2.1355	<0.005*
*Cbuq 67219_g1*	2,394.3122	1,089.8032	1.1355	<0.005*
*Cbuq 67727_g4*	682.3091	612.6378	0.1554	>0.005
*Cbuq 74007_g1*	205.409	2,545.3153	–3.6313	>0.005
*Cbuq 74056_g1*	9.2572	6,088.6258	–9.3613	<0.005*
*Cbuq 7577_g1*	195.0692	10.4332	4.2247	<0.005*
*Cbuq 85742_g1*	779.0916	4,956.5446	–2.6695	<0.005*
*Cbuq 97345_g1*	675.4117	171.5792	1.9769	<0.005*
*Cbuq 97376_g1*	17.9691	1,310.7893	–6.1888	<0.005*
*Cbuq 97535_g1*	146.9063	5,731.4302	–5.2859	<0.005*

Q values were calculated according to the method of Anders et al., 2003.

*q < 0.005 is significantly different.

**Table 4:** Differentially expressed PBPs between females and larvae.

Gene	Readcount-Female	Readcount-Larvae	log_2_Fold-change	q
*Cbuq 12614_g1*	1,264.2533	4,519.3072	–1.8378	<0.005*
*Cbuq 15552_g1*	75.6752	290.9021	–1.9426	>0.005
*Cbuq 16395_g1*	1,059.9728	10,400.0016	–3.2945	<0.005*
*Cbuq 25979_g1*	1,621.9646	3,791.0921	–1.2249	<0.005*
*Cbuq 29237_g1*	12.3564	242.5871	–4.2952	<0.005*
*Cbuq 37516_g1*	9,632.1227	34.4349	8.1278	<0.005*
*Cbuq 46750_g1*	528.8098	459.7396	0.2019	>0.005
*Cbuq 47175_g1*	35,808.2596	42,171.6927	–0.236	>0.005
*Cbuq 61968_g1*	2,660.9358	1,038.9972	1.3567	>0.005
*Cbuq 61968_g2*	2,795.9667	4,953.4406	–0.8251	>0.005
*Cbuq 67219_g1*	1,399.4825	1,211.938	0.2076	>0.005
*Cbuq 67727_g4*	881.4039	681.0994	0.3719	>0.005
*Cbuq 74007_g1*	606.8011	2,841.9673	–2.2276	>0.005
*Cbuq 74056_g1*	12.5463	6,754.1377	–9.0724	<0.005*
*Cbuq 7577_g1*	280.7007	11.6208	4.5943	<0.005*
*Cbuq 85742_g1*	1,247.8847	5,512.1001	–2.1431	<0.005*
*Cbuq 97345_g1*	799.4617	190.8162	2.0668	>0.005
*Cbuq 97376_g1*	5.5456	1,454.4069	–8.0349	<0.005*
*Cbuq 97535_g1*	130.7661	6,357.5929	–5.6034	<0.005*

Q values were calculated according to the method of Anders et al., 2003.

*q < 0.005 is significantly different.

**Table 5:** Differentially expressed PBPs between males and females.

Gene	Readcount-Male	Readcount-Female	log2Fold-change	q
*Cbuq 12614_g1*	1,252.1442	1,302.6029	–0.0570	>0.005
*Cbuq 15552_g1*	129.7305	78.0688	0.7327	>0.005
*Cbuq 16395_g1*	801.3284	1,092.1459	–0.4467	>0.005
*Cbuq 25979_g1*	1,461.5281	1,672.1981	–0.1943	>0.005
*Cbuq 29237_g1*	13.7319	12.7262	0.1097	>0.005
*Cbuq 37516_g1*	7,846.6603	9,921.5929	–0.3385	>0.005
*Cbuq 46750_g1*	693.3326	544.2083	0.3494	>0.005
*Cbuq 47175_g1*	17,972.7056	36,783.4231	–1.0332	>0.005
*Cbuq 61968_g1*	1,689.2178	2,737.3336	–0.6964	>0.005
*Cbuq 61968_g2*	1,112.7737	2,871.6207	–1.3677	>0.005
*Cbuq 67219_g1*	2,626.2247	1,443.8784	0.8630	<0.005*
*Cbuq 67727_g4*	748.7749	909.5280	–0.2806	>0.005
*Cbuq 74007_g1*	225.8287	623.8702	–1.4660	<0.005*
*Cbuq 74056_g1*	10.1648	12.9063	–0.3445	>0.005
*Cbuq 7577_g1*	647.3061	223.6264	1.5334	<0.005*
*Cbuq 85742_g1*	856.4596	1,284.5830	–0.5848	>0.005
*Cbuq 97345_g1*	741.3466	821.6198	–0.1483	>0.005
*Cbuq 97376_g1*	19.5857	5.7071	1.7790	>0.005
*Cbuq 97535_g1*	161.0591	134.2881	0.2623	>0.005

Q values were calculated according to the method of Anders et al., 2003.

*q < 0.005 is significantly different.

### Validation of transcriptome data

**Fig 10:**
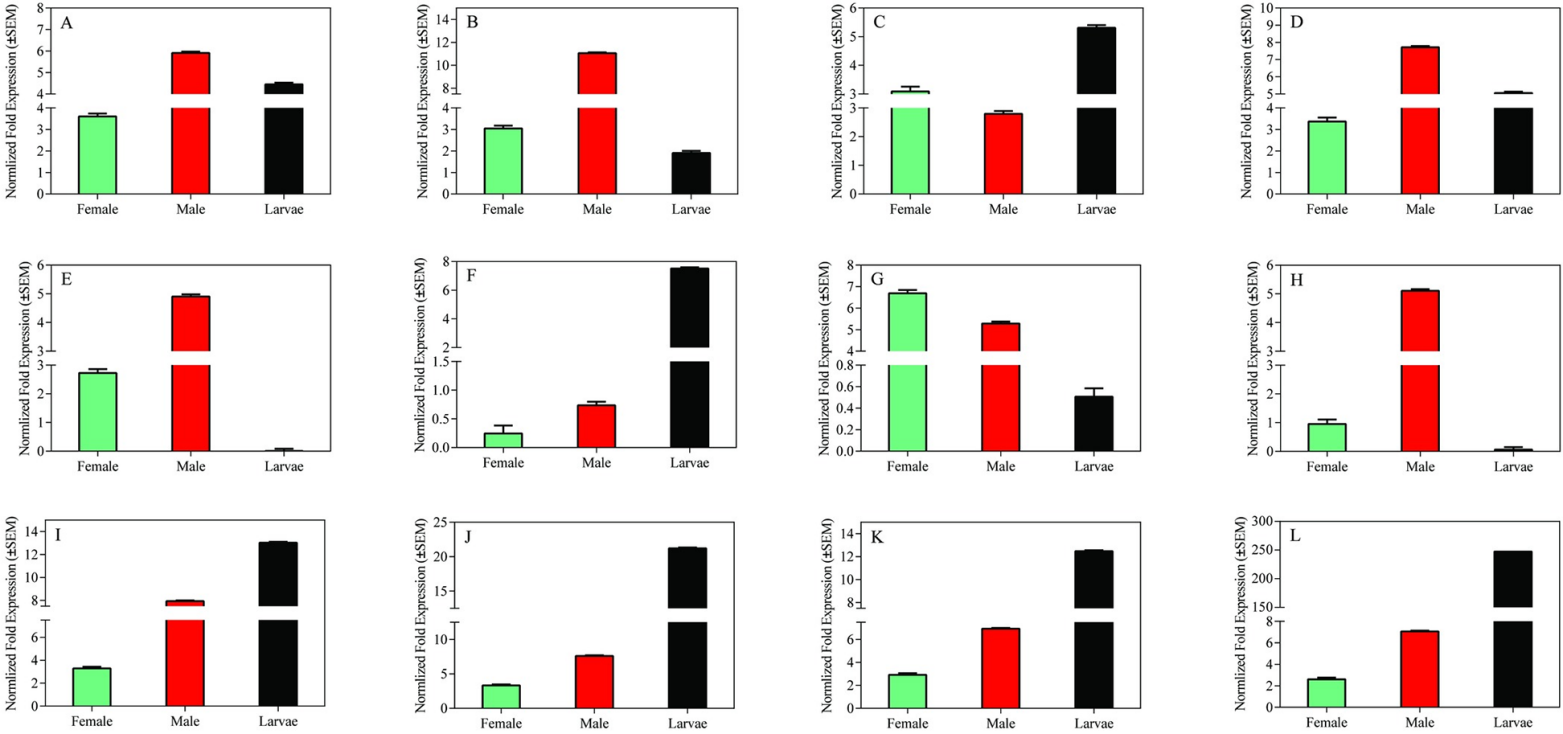
qPCR results of differentially expressed genes in larvae, male and female adults. The expression levels of the mix-aged larvae, male and female adults were showed by black, red and green bar, respectively by the results of 2^-ΔΔCT^ method with three biological repeats. Sub-caption A to L indicate the identified different expressed genes between the larvae, male and female adults (A: *Cbuq12614_g1* B: *Cbuq16395_g1* C: *Cbuq25979_g1* D: *Cbuq29237_g1* E: *Cbuq37516_g1* F: *Cbuq74007_g1* G: *Cbuq67219_g1* H: *Cbuq7577_g1* I: *Cbuq85742_g1* J: *Cbuq97376_g1* K: *Cbuq97535_g1* L: *Cbuq74056_g1*)

**Fig 11:**
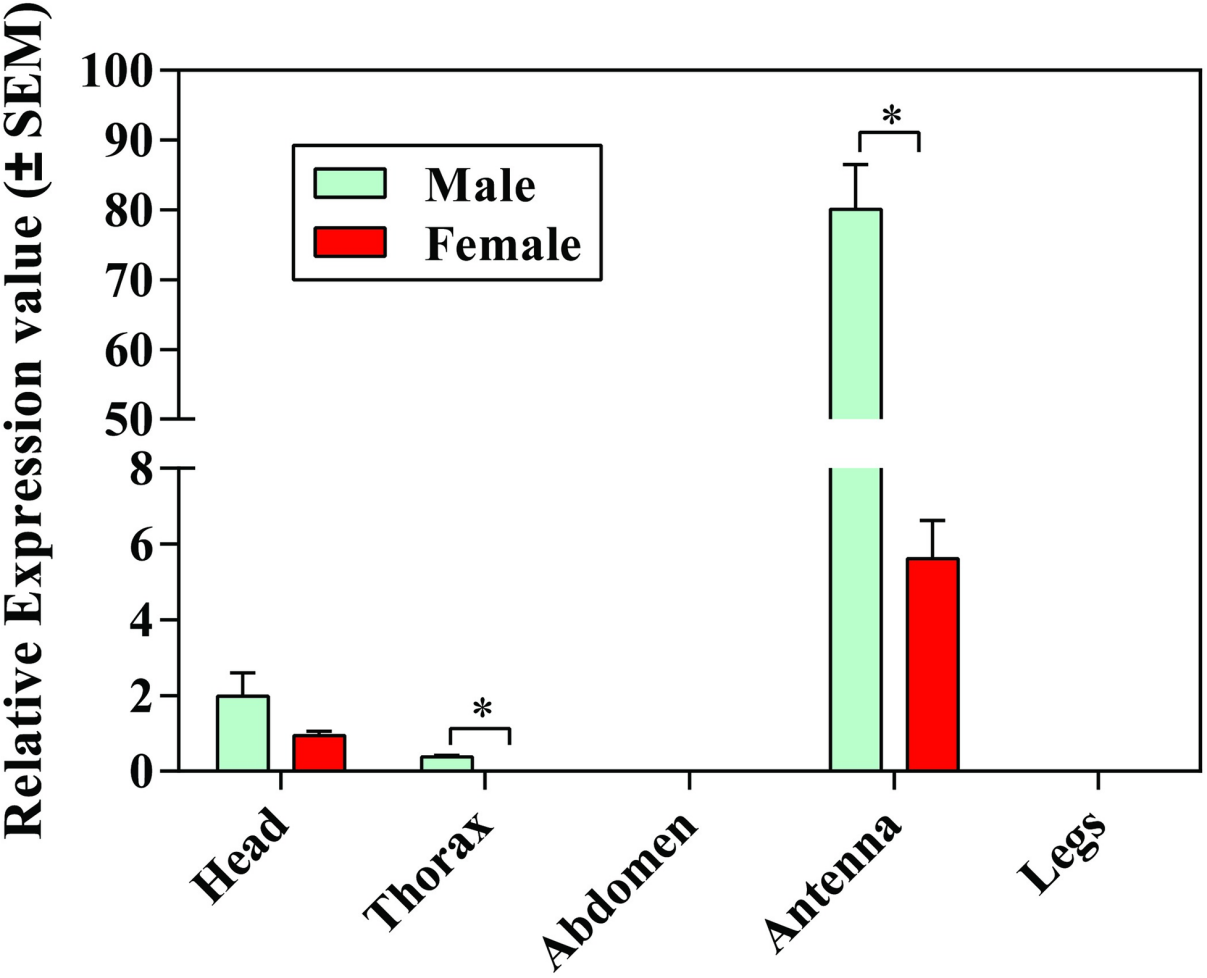
Tissue- and sex-dependent expression patterns of *Cbuq7577_g1*. The expression levels of *Cbuq7577_g1* in various tissues are shown for males (green) and females (red) based on the 2^-ΔΔCT^ method for three biological repeats.

To validate the transcriptome result, we selected 12 significant differentially expressed genes from Tables 3–5 for quantitative reverse transcriptase-PCR (qRT-PCR) confirmation. Six PBPs transcripts which have demonstrated by RNA-seq to be enriched in larvae were confirmed by qRT-PCR (Fig 10) (S31 Text). Additionally, *Cbuq7577_g1* had significantly higher transcriptional level in male than in female and larvae with 5.36 and 85.19 fold exchanges. Moreover, to further explore tissue- and sex-specific expression, we selected *Cbuq7577_g1* for qRT-PCR confirmation. We observed the highest expression of PBP *Cbuq7577_g1* in male antennae, followed by male heads, compared with low levels of expression in female antennae and heads (Fig 11).

## Discussion

### Overview of transcriptome data

The transcriptome is the complete set of expressed RNA transcripts in one or more cells [32], and its analysis enables the study of gene transcription and the characteristics of transcriptional regulation. High-throughput sequencing technology has been applied to the transcriptome study of many species in class Insecta, such as *Phyllotreta striolata* [33], *D*. *melanogaster* [34], *Biston betularia* [35], *Aedes aegypti* [36], *Brugia malayi* [37], and *Bemisia tabaci* [38].

In the present study, developmental transcriptomes were established of *C*. *buqueti* eggs, mixed-age larvae, pupae, and male and female adults, providing a relatively comprehensive gene pool. The numbers of clean reads from egg, larval, pupal, female and male transcriptomes were 31,469,916, 36,773,825, 32,128,345, 33,070,448, and 31,434,121, respectively. All these clean reads were assembled into 108,854 transcripts by Trinity software. A total of 83,115 unigenes were annotated by Nr, Nt, KO, Swiss-Prot, PFAM, and KOG/COG. Thousands of differentially expressed genes were identified, which facilitates developmental and evolutionary studies of *C*. *buqueti*, and contributes to future work in bamboo snout beetle comparative genomics.

### Pheromone-binding proteins

Previously reported physiological functions of PBP include: binding specificity, transporting specific pheromone molecules, and filtering odorant molecules entering the sensor chamber [39]; acting as a carrier to transport pheromones through the hemolymph to the receptor [40]; forming the PBP–pheromone complex for receptor recognition [41], cascade initiation, and deactivation to restore receptor sensitivity [42]; and protecting pheromones from enzymatic degradation [41]. Identifying the developmental transcriptome of *C*. *buqueti* provides an opportunity to understand the physiological function of *PBPs*. A total of 19 candidate *PBPs* were identified in the present study.

The alignment of *CbuqPBP1* and 27 known coleopteran insect olfactory gene sequences, and a phylogenetic tree indicated that *CbuqPBP1*, *CmonOBP2* and *CbowOBP26* are on the same clade. Therefore, it was speculated that such genes may have the same ancestral gene, but were differentiated by adaptation to different types of environmental chemical factors during evolution, and perform the same or similar functions among different species.

Thirteen candidate PBPs in male adults and larvae (Table 3), 10 candidate PBPs in female adults and larvae (Table 4), and three candidate PBPs in female and male adults (Table 5) showed significant differences in expression, with *Cbuq7577_g1* demonstrating significant differences in expression among larvae, male adults, and female adults. *Cbuq7577_g1* showed 49% identity with the OBP of *Dendroctonus ponderosae* (AGI05175), and 47% identity with the OBP of *Lissorhoptrus oryzophilus* (AHE13794). AGI05175 and AHE13794 were previously functionally annotated as insect pheromone/OBP domains. *Cbuq7577_g1* in *C*. *buqueti* showed very low similarity to genes in the NCBI database, which likely reflects the limited research that has been carried out into Curculionidae and Lepidoptera PBPs. To research gene function, a PBP gene of *C*. *buqueti* (*CbuqPBP1*) was cloned in this study for prokaryotic expression. Using N-phenyl-1-naphthylamine as the fluorescent probe in a competitive binding assay, the ability of *CbuqPBP1* to bind 12 sex pheromone analogs and three volatiles of *Neosinocalamus affinis* shoots was examined. These results will be published later.

qRT-PCR results of the present study showed that candidate PBP *Cbuq7577_g1* in *C*. *buqueti* is expressed in male and female adult antennae, which is consistent with the expression pattern of PBPs in most insects, such as *Heliothis virescens* [43], *Manduca sexta* [44], *Spodoptera exigua* [45], and *B*. *mori* [46]. PBP expression in adult females may enable the identification of hydrophobic pheromones in the male or the monitoring of pheromones released by the female, as well as transporting pheromones and general odorant molecules. Although it is generally believed that insect PBPs are only expressed in the antennae, researchers have also documented their expression in the head, feet, wings, and other body parts [46, 47]. Zhang et al. [48]found *HarmPBP2* of *Helicoverpa armigera* was expressed in female’s maxillary palp, but the highest expression in the antennae. The candidate PBP *Cbuq7577_g1* was mainly expressed in antennae (97.07%). Its expression level in male antennae was 14.43 times that in female antennae. *Cbuq7577_g1* may play an important role in the identification of odorant molecules, specifically those involved in identifying females from the external environment through *C*. *buqueti* antennae.

## Conclusions

We constructed a comprehensive, good-quality sequence resource from the developmental transcriptomes of *C*. *buqueti* eggs, larvae, pupae, and female and male adults. This resource enriches what is known about *C*. *buqueti* genomics, thus facilitating our understanding of metamorphosis, development, and fitness to environmental change. The identified candidate PBP *Cbuq7577_g1* might play a crucial role in identifying sex pheromones, and could be used as a target to control *C*. *buqueti* as a pest by disrupting sex pheromone communication.

## Supporting information

S1 Text Eggs1 annotation. (XLS)

S2 Text Eggs2 annotation. (XLS)

S3 Text Eggs3 annotation. (XLS)

S4 Text Female1 annotation. (XLS)

S5 Text Female2 annotation. (XLS)

S6 Text Female3 annotation. (XLS)

S7 Text Larvae1 annotation. (XLS)

S8 Text Larvae2 annotation. (XLS)

S9 Text Larvae3 annotation. (XLS)

S10 Text Male1 annotation. (XLS)

S11 Text Male2 annotation. (XLS)

S12 Text Male3 annotation. (XLS)

S13 Text Pupae1 annotation. (XLS)

S14 Text Pupae2 annotation. (XLS)

S15 Text Pupae3 annotation. (XLS)

S16 Text Annotation GO. (XLS)

S17 Text Annotation KEGG. (XLS)

S18 Text Annotation KOG. (XLS)

S19 Text CE vs CP. DEG. (XLS)

S20 Text CF vs CE. DEG. (XLS)

S21 Text CF vs CL. DEG. (XLS)

S22 Text CF vs CP. DEG. (XLS)

S23 Text CL vs CE. DEG. (XLS)

S24 Text CL vs CP. DEG. (XLS)

S25 Text CM vs CE. DEG. (XLS)

S26 Text CM vs CF. DEG. (XLS)

S27 Text CM vs CL. DEG. (XLS)

S28 Text CM vs CP. DEG. (XLS)

S29 Text Deduced amino acid sequences of *Cbuq7577_g1*. (DOCX)

S30 Text Deduced amino acid sequences of PBP genes. (DOCX)

S31 Text The Genbank number and open reading frame of all identified candidate PBP genes. (DOCX)

S32 Text The dataset and accession number. (DOCX)

S1 Table Transcriptome software and parameters list. (DOCX)

S2 Table Nucleotide sequences of all identified candidate PBP genes. (DOCX)

S3 Table Primer used in RT-PCRs and qRT-PCRs. (DOCX)
