# Supplementary material for: The developmental transcriptome of the bamboo snout beetle Cyrtotrachelus buqueti and insights into candidate pheromone-binding proteins
Source: PLoS One. 2017 Jun 29;12(6):e0179807. doi: 10.1371/journal.pone.0179807 (PMC5491049; doi:10.1371/journal.pone.0179807)
Supplement: S29 Text — (DOCX) [file pone.0179807.s029.docx]

>c7577-g1

MKLLLVLALALVAVNGLSESLVDEMKEKLQKYGLECAEKEKASEEDIQALMNHERPVTHAGKCTIFCTFKKFDLM KEDGSFGPGDMDWIERAKADDAEFMEKLTGIQSTCEKTVQIDSDPCETALRAAKCAKDEGEKLGITSF
