# Supplementary material for: The developmental transcriptome of the bamboo snout beetle Cyrtotrachelus buqueti and insights into candidate pheromone-binding proteins
Source: PLoS One. 2017 Jun 29;12(6):e0179807. doi: 10.1371/journal.pone.0179807 (PMC5491049; doi:10.1371/journal.pone.0179807)
Supplement: S30 Text — (DOCX) [file pone.0179807.s030.docx]

>c12614_g1;orf1 len=155 frame:-2 start:931 end:467 gi|332374168|gb|AEE62225.1| unknown [Dendroctonus ponderosae]&gt;gi|478258063|gb|ENN78201.1| hypothetical protein YQE_05353, partial [Dendroctonus ponderosae]&gt;gi|546682562|gb|ERL92485.1| hypothetical protein D910_09798 [Dendroctonus ponderosae]&gt;gi|828177629|gb|AKK25138.1| odorant binding protein 13 [Dendroctonus ponderosae]

LQLASGPNMNGLLKVSVLIIVVSAISCQEFTEEQKKKILENRKQCIEESKVNPELIEKAD

QGNFAEDTSLKCFTKCFYQKAGFVNDEGEVQLDVVKAKLPAQADKEQALAIVEKCKIKGK

DACDTVYLIHKCYFEHTHPELFKKDEPKKEEKKA*

>c15552_g1;orf1 len=155 frame:-3 start:717 end:253 gi|471180457|gb|AGI05179.1| odorant-binding protein 26 [Dendroctonus ponderosae]&gt;gi|478251606|gb|ENN72068.1| hypothetical protein YQE_11354, partial [Dendroctonus ponderosae]&gt;gi|546674285|gb|ERL85696.1| hypothetical protein D910_03111 [Dendroctonus ponderosae]

IVMKNSLIGAFVLCTWLLDYVQLAPPPTFQLPSEEERNRIALKCIDEVMIEKNIIEEVLK

TQVLPHDDQKYKKFLECSYRKQGFLSLDGSRMLYDNLFLFLSEFYEIEDLDALQHCKFIK

SKDAGDLCFQNLSCILDALRTVETLNGEDENNVQ*

>c16395_g1;orf1 len=169 frame:-2 start:562 end:56 gi|568599606|gb|AHE13798.1| odorant binding protein [Lissorhoptrus oryzophilus]

RRSNDISVPISVSENYSNKTMNQLTVVVFFACVAALLASPLEPKAAAAQASQDRLKAAHH

KCQSNPATAVEESALKALTSGGPKPANYGAHALCMSKALGWQSEDGSVNTETIKARAENI

FGPSPKLNEIVNECAQNQANAEETAVHLTRCYVKYAPRHNGHPPGHPH*

>c25979_g1;orf1 len=282 frame:-2 start:7609 end:6764 gi|332373184|gb|AEE61733.1| unknown [Dendroctonus ponderosae]

LFTKMASPKVAVVTGSNKGIGFAIVKGLCEKYDGQVYLTARNVQRGEAAVEALKKLGYKP

LFHQLDITDQNSVDAFKEYLKQKHGGIDVLVNNAAIAFPGNSEESFGTQAKETIRVNYFA

TLRICEALFPLLRQNARVVNVSSMMGHLSKIPSQTLQEQLKSDKLTIAELNQLMEKFVRD

SEAGKNVEEGWGSSAYAVSKVGVSALTFVQQRLFDAETPNRNIAVNAVHPGYVDTDMTSH

RGVLTPEQGARAPLFLALEATFKGKYVWDDTKVVDWIKPLI*

>c29237_g1;orf1 len=359 frame:-1 start:1356 end:280 gi|478259671|gb|ENN79515.1| hypothetical protein YQE_03978, partial [Dendroctonus ponderosae]&gt;gi|546682941|gb|ERL92820.1| hypothetical protein D910_10128 [Dendroctonus ponderosae]

PSYQIKMKYNLGLLVLVVYVNVITAFGSSEDRFIKKYAMMKIYESCFGPDVVRQIRHEMK

AACAKCASYETPPTTPAPQSTTEEVQQADTPTANTGFPNNNAPLDVEKLHQAIMAYRPNV

PQAALRPAYPQNLNNFYSPVAYTNPGFQSGGLPFFYPGYQQIPFSPYGGFPVVGQQFYPG

SHRMSRDMDVRAQIEAITSRMSGKVKNVTCVMQELGYLDDNLEPNFSKISERIGNLPVDD

ELKRDMQDGVSFCKQFSQCVPEVNKDKSPLSRELIRPMFFFKCYKHKKLEACVMKDVREK

YAGVSDDFENGDIELRRTGRQGKAGKIDDKEKEIDDLASSMYDFLYTSDSGFDVDGIL*

>c37516_g1;orf1 len=143 frame:-1 start:564 end:136 gi|568599592|gb|AHE13791.1| odorant binding protein [Lissorhoptrus oryzophilus]

LPIIKMKQVLALVLCVSVLIMGQSVLERWEKLHDICQADPATFVDESIFKKIKDNETNVE

LPPNFGAHVFCMTVNLNIQDPNGKFNKEVTAKLIGEVVKDQAKVNKVVNECAVNKPNKDD

AAVAFLQCLDRNNVDIGQRETY*

>c46750_g1;orf1 len=138 frame:-1 start:969 end:556 gi|471180463|gb|AGI05182.1| odorant-binding protein 29 [Dendroctonus ponderosae]&gt;gi|478258066|gb|ENN78204.1| hypothetical protein YQE_05356, partial [Dendroctonus ponderosae]

LFKMKALLAIVALISAAVYVTVFAFTLLSEGKEFGDEVVKQCITETSISKDILDMDTINE

ENRDKVGSFALCVSKKVGYQDDDGNLQTDAIKKALTSSVGNTDQVNTLMRKCFVQKSQAK

ETALASLLCFSDELSSN*

>c47175_g1;orf1 len=141 frame:3 start:303 end:725 gi|828177622|gb|AKK25135.1| odorant binding protein 9, partial [Dendroctonus ponderosae]

YDVNIPTMKTFIVLSAFVLAVVLADTTKSSWNRVHKACQAKPGVFVDDAIFEKLKRNEKV

TLPANFGVHAHCMLEGFGIQNSQGAIQQNGIKKAVQESVSDPAKANQIVSACSVSKSSKE

ETALGIFNCFGKNSVDIGQF*

>c61968_g1;orf1 len=144 frame:-2 start:1149 end:718 gi|828177622|gb|AKK25135.1| odorant binding protein 9, partial [Dendroctonus ponderosae]

NRSLQEVTMKVLCLALFLLAAIVLGDEVQDRYDNVHKGCQKDPALYVDDAIFAKLKRGEK

VNNLPANFGAHAFCMLKNLDLQDSQGKIQQAAVQKAVERSEADQVKAKRITAECSALNKG

TKEDSALALFDCLGKNRINIGQL*

>c61968_g2;orf1 len=154 frame:2 start:212 end:673 gi|568599594|gb|AHE13792.1| odorant binding protein [Lissorhoptrus oryzophilus]

RLTAMKIFVAFSAVLFVVLAEHQHEHAHQHHPEDVHGLAKVHTVCQSSDSTYVDNDVFQK

LDRNVPVVLPANFGKHLLCMMKGIGTVSADGQPNVEGIKTHIHHVIHDESKAAHILRECA

VAKNTPEQTSIDLEACLTKHHVFGGPAEHHHHP*

>c67219_g1;orf1 len=168 frame:3 start:6933 end:7436 gi|546686045|gb|ERL95445.1| hypothetical protein D910_12708 [Dendroctonus ponderosae]

SYPSELLEVVIKGVPSMHICLDFLQELMQQPSLTKQIFAVQLLSHLCVQYALPKSLNLCV

TALNLLYALLGGISSVQRVKLFKPVLPALVRISEAFPPLTDDIVNLLMQLARICESQASL

ASHFDSQRGKGLEISAQESAELCDLTKKTFTEILDKTVLRTNVYRQE*

>c67219_g1;orf2 len=819 frame:-1 start:10711 end:8255 gi|546686045|gb|ERL95445.1| hypothetical protein D910_12708 [Dendroctonus ponderosae]

KNSIHILCSLLYIVQKRQLHIENKLNIMEFEDELLDEDNSEFFINFPPEVQKAIDEVLPN

RDSLDEPDFNSIDYINSLFPTEQSLSNIDEMVIKMENQISTIDNEISTVIRSQIEASSDG

REALDEAQKIIKQLFLHIKDIKERAEKSEEMVREITRDIKQLDCAKRNLTLAITTLNHLH

MLVGGVDTLKSLTQKRLYGEIALPLQAISEVMTHFENYSDIPQIKNLSDQVKSIHVELAE

QITHDFKEAFEGTNSKNIVPNKQLQQACLVVSILNPKVKRELLKWFVGLQLQEYNHLFQE

TEDTAWLDKIDKRYAWIKRHLLEFEDRMGNMFPQNWEVSERIVVQFCHNTREELAKIMSK

RKGEIDVKLLLYAIQKTSAFENLLFKRFTGITLRENLDSNEKLKAENKKGEKNVSNPQLD

FNESLFNGLIGQCFITHLEIYIESLDRNLADLIDRFVQDEKQHRPIENAETQAAILSSCP

DLFVFYKKSMIQCTQLDKGQSMLSLTRIFQKYLQEYSEKLLYNNLPKIEGQSLGSSVQNF

TKDLQKMSTSGLIQNFSSLLKEGEVIRFTKDEQTKICCILTTAEYCLETTQQLQDKLKEK

IDPLLADQIDLSKEQDQFHKVISNCIQLLVQDLENACEPSLTAMSKIPWQNIDAVGDQSP

YITSITTHLKTTIPIIRDNLAHSRKYFTQFCIKFANSFIPKFIQSIYKCKPINTVGAEQL

LLDTHMLKTVLLNLPSISSQINRPAPTAYSKVVTKGMTKAEMILKVVMTPIEPQKNFIDQ

CKKLIPDCQLTEFHKILDMKNIKRQEQAVLVDLFKYTK*

>c67219_g1;orf3 len=970 frame:1 start:3370 end:6279 gi|546686045|gb|ERL95445.1| hypothetical protein D910_12708 [Dendroctonus ponderosae]

IYCINMTFKKCVNPVVFKAVQNVDMEKLAKCSEGNIRPVLPCLVRMGLISPLDTSRACTN

MKVNILTVVSGMELVNSIVALLSIDFHKLEIEVKKEQQLRQKGNSSQNDSILIGNLSNTS

MALEYERSDMTRRLSILLGELLFIQSQIQELPETPDAETYIKSSELFDNDIFAEELSDII

CIALAELPTTLNISNIVETLLHVHNGPEIICRVVANFPDCFREVCTYLIQTGEKQEESIS

STIRSTTIGLLCQMNPSQSLSVRSKCVELCRMPALAIALSLGDICGDSDGDMVAFISGLL

LGNDQTIRNWIAMFIRTGQKRKGEASSNALQQLREELLKRLQKIIDFSPEGQIPDSLVVQ

ASALLRLYCALRGIAAIKFQDEEVNLLVQLLTSHPNPTPAGVRFVSIGLCMLIACPSLIS

QPEHERRSIEWVQWLVKEEAYFESASGVTASFGEMLLLMAIHFHSQQLSAICELTCATLG

MKIAIRHNNMNRMKQVFTQEIFTEQVVTAHAVKVPVTQGLSANMTGFLPIHCIHQLLKSR

AFAKHNVNIKNWIYKQICSSISPLHPVLPMLVEVYVNSIMIPNSKNLEQANKPLTENEIR

RVFQSSIFGQYFENKQSIFTMEFDVNFENQDVVVDNTSLTPQLLLLYYLLLYEDCRLNNA

HLLASSGKKIKQYTSEFMSELPIKYLLHHAQKDQSSYSGLFGPLLKLLATHFPHLTLVED

WLDDMSIQTEKKLVQIDEYMVVAAFNEIETNPSKCAKLLQTMLKIEAIDIWPFAESFTQF

ARNVLGENIPRFVQDLYKDVWLRLNTVLPRRLWALTIKNLVDDFSSITKIDVAEDPLQIM

RCDERVFRCAPVYAIVLRVLRASLASSRSQLTQHLQSNPKLDSHGQILNEPDREEMCRAT

TAAQESAAVQMLLETCIENYLDKTTPGRQWALQEVRSLVCSYLHQVFIADTMLCKLVHFQ

VSKSINCNM*

>c67727_g4;orf1 len=463 frame:1 start:2749 end:4137 gi|332375190|gb|AEE62736.1| unknown [Dendroctonus ponderosae]

NMSRQSVLQPVDVNVDHSPARKRARFDENVDDSNKSNLWILSNKELVLHEIYDHGIGRLD

CDSDDVVTEDDSIFNKTEGIGIDVSCASMTPNSRGKQLSILESTLTKESLDKLSNGEWDN

LFNKTSIKPTDKTNYFDELSDEVILQVFRWLPKSYLGDIALVCRRFYRLTQDESLWTRMD

VSNKHLAAGELGKILSKQVVVLRLARSEIMHKPILPGCKANFSDFRSRLIYLDLSMAHIS

LDSLVMLFNKCRRLKKISLENVPVSDDVLIALSANKDIEVINFAMCTGMQEDGLRYLLTN

CRQIRELNIAWTYLNGDCIEYLCENLPSSMDRLNFSGCRKLLLDKHVASLVLSCPRLREL

DLSDCTSITGESVRQITVLEDLAFVALSRCYLIPYKSLGQLRKLINLSYLDIHGGYIDGE

ELKEVQNDLGAGVQINQFKFSSVARPTVGMRRSSIWNMRVRD*

>c67727_g4;orf2 len=281 frame:-3 start:2543 end:1701 gi|332375190|gb|AEE62736.1| unknown [Dendroctonus ponderosae]

INLCDITFKMSRILTNIYQKLNITLLPHYSSKFKFPAVYFDRRIRCYSQKRLPKHPSYDL

SKGIGPITWRSLGITAGLGATLLAFMLYVKKEKEIALDKERNRMLGKAAIGGKFELVDSL

GKLRNSDEFLGQWLLIYFGFTHCPDICPDELEKMSDVIDNLDKEKNVSKIQPLFISVDPN

RDTPEIVGKYCKEFSPRLLGLTGSEEQVAKTCKAYRVYFSTGPKDKDKDYIVDHTIIMYL

VNPDGSFVDYYGQNRTSSEIASSIKLHISKYNYNKNSWFS*

>c74007_g1;orf1 len=139 frame:-1 start:519 end:103 gi|471180455|gb|AGI05178.1| odorant-binding protein 28 [Dendroctonus ponderosae]&gt;gi|478258071|gb|ENN78209.1| hypothetical protein YQE_05361, partial [Dendroctonus ponderosae]&gt;gi|546682567|gb|ERL92490.1| hypothetical protein D910_09803 [Dendroctonus ponderosae]

KITVNMKWSIAILLCFILSTVLALTVEESKEKVKKAHEKCNGDAATKLDPEEKKAYKSSK

VVGPSLKAHALCVSKTLGWQHPDGKIDKTSVKEKISSFITDKEQADKIYSECLVDHDDEK

DTAHNLLVCYGRHFGHKH*

>c74056_g1;orf1 len=141 frame:1 start:19 end:441 gi|568599608|gb|AHE13799.1| odorant binding protein [Lissorhoptrus oryzophilus]

GYRCYSDKMKYFILISVLVSVFTCGFAASRATWTQKFFSFTNECIADTGIEADIVQKALQ

GHITNDPKLKTFLFCMTKKGALQNANGEVQIEEFKKQLPSLVENPETTIELVRKCVWKEG

TPEDIALQIYGCFYKTDSNK*

>c7577_g1;orf1 len=168 frame:1 start:67 end:570 gi|471180449|gb|AGI05175.1| odorant-binding protein 8 [Dendroctonus ponderosae]

ENPSIDSQSFIQQCCYIASVLHCNMKLLLVLALALVAVNGLSESLVDEMKEKLQKYGLEC

AEKEKASEEDIQALMNHERPVTHAGKCTIFCTFKKFDLMKEDGSFGPGDMDWIERAKADD

AEFMEKLTGIQSTCEKTVQIDSDPCETALRAAKCAKDEGEKLGITSF*

>c85742_g1;orf1 len=173 frame:2 start:251 end:769 gi|471180469|gb|AGI05185.1| odorant-binding protein 9 [Dendroctonus ponderosae]&gt;gi|478258070|gb|ENN78208.1| hypothetical protein YQE_05360, partial [Dendroctonus ponderosae]&gt;gi|546682566|gb|ERL92489.1| hypothetical protein D910_09802 [Dendroctonus ponderosae]

LSIHILRYKLEEKVLKNVYFYLFFGLSCFGCFSGCVKMKFVVLFCVVLLLATVVSSKKHH

KNNNEVTPKKAFKECQKNATTRIDKQAVKKYKKKEVDSMPQNYGEHLLCIYKATGYIGED

GVVNQDVLKKKITKKAQPSQNVDTLLQECGAAKADPKQTAINLDSCLTKNNL*

>c97345_g1;orf1 len=138 frame:2 start:50 end:463 gi|471180451|gb|AGI05176.1| odorant-binding protein 30 [Dendroctonus ponderosae]&gt;gi|478258065|gb|ENN78203.1| hypothetical protein YQE_05355, partial [Dendroctonus ponderosae]

INKMKLLIFAVFLLVTLFQVKCQTDKQKELLAQHYKQCVEQTHVDQNVLQQARAGNFTDD

PKLKDHILCITKKIGFQDEAGHLQKEVIQKKLKEAVKGNEDQTKKLMEACAITNQDPKLQ

AFNAFKCIHHKAKINLL*

>c97376_g1;orf1 len=267 frame:1 start:130 end:930 gi|471180415|gb|AGI05158.1| odorant-binding protein 2 [Dendroctonus ponderosae]

YLLAIIMSGLLALCAILATALAITVAYDFEDADFNQFLADDLEDGLDTLDSAFVHYRVRR

AEDANPPAQSGDDKCKKRRRKPSLCCADDIIDQQHEKDRETFRSCFREVLGVEKSGHHRR

GDPFSCKEAEKRRNDMTCVTTCYGQKKGFLDDQGNPIPEALTKSLKDAFAKESWFDGVAD

KIVTTCLKEADNATQYQPKPSSDDIKLCNPSGLTLKHCLFKQIQLSCPADQIKDQKACDK

FQDRIKKGLDDVEPQPPPPFDGPRDD*

>c97535_g1;orf1 len=147 frame:-1 start:513 end:73 gi|471180463|gb|AGI05182.1| odorant-binding protein 29 [Dendroctonus ponderosae]&gt;gi|478258066|gb|ENN78204.1| hypothetical protein YQE_05356, partial [Dendroctonus ponderosae]

ELNNKQNRSKMKSLLVISTILCVFIYVLADLSDEQKQKVLNYGKECIAETGVDKELVLKA

RQGSFSDDPKLKAFAFCLSKKIGLQNASGDVQHEVLKEKLSSVVDNAETVNSLISACVQN

KGSPEETAYQTFVCYYEKTPSHASIF*
