# Supplementary material for: The developmental transcriptome of the bamboo snout beetle Cyrtotrachelus buqueti and insights into candidate pheromone-binding proteins
Source: PLoS One. 2017 Jun 29;12(6):e0179807. doi: 10.1371/journal.pone.0179807 (PMC5491049; doi:10.1371/journal.pone.0179807)
Supplement: S31 Text — (DOCX) [file pone.0179807.s031.docx]

No. KU845733 Cbuq_7577

ATGAAGCTTTTACTGGTCTTGGCTTTGGCGCTGGTAGCTGTCAATGGGCTTAGCGAAAGCTTAGTTGATGAGATGAAAGAGAAGTTACAAAAATATGGATTGGAATGTGCTGAAAAAGAGAAAGCATCAGAAGAGGATATCCAAGCTCTGATGAACCACGAACGACCAGTCACCCATGCCGGAAAATGCACTATTTTCTGTACATTCAAAAAATTCGATTTGATGAAAGAAGATGGATCCTTTGGTCCCGGTGACATGGACTGGATTGAAAGAGCCAAAGCTGACGATGCGGAATTCATGGAAAAACTAACGGGTATACAATCCACATGTGAAAAGACAGTTCAAATAGACTCTGACCCGTGTGAGACCGCATTACGAGCTGCAAAATGTGCTAAAGATGAAGGCGAAAAACTTGGAATTACCAGTTTTTAA

No. KX814422 Cbuq_12614

ATGCGTGTGTTCGAAATAGCACTTGTGTATCAAATAAACGGTATCGCACGCGTCCTTTCCTTTAATTTTACATTTCTCCACGATTGCGAGGGCCTGTTCTTTATCGGCCTGCGCCGGCAGTTTGGCCTTTACGACGTCCAACTGTACTTCGCCCTCGTCATTCACGAATCCCGCCTTCTGGTAGAAACACTTGGTGAAACATTTGAGGGAGGTGTCTTCCGCGAAGTTTCCTTGGTCCGCTTTCTCGATCAGTTCCGGATTGACTTTGCTCTCCTCAATGCATTGTTTACGGTTCTCGAGGATTTTCTTCTTTTGCTCTTCGGTGAATTCCTGGCATGA

No. KX814423 Cbuq_15552

ATGAAAAACTCGCTCATTGGTGCATTTGTTTTGTGTACATGGCTACTGGATTA TGTACAGCTT GCTCCACCAC CTACTTTTCAACTTCCCTCCGAAGAAGAAAGAAATCGCATAGCTCTGAAATGCATCGACGAGGTGATGATTGAAAAAAACATAATCGAGGAGGTTCTCAAGACTCAAGTACTTCCACACGACGACCAAAAGTACAAGAAATTTCTTGAGTGTAGCTACAGGAAACAGGGCTTTCTTTCACTGGACGGTTCTCGGATGCTCTACGATAATTTGTTCCTGTTCTTGTCTGAGTTTTACGAGATCGAAGACTTAGACGCGCTCCAGCACTGCAAATTTATCAAGTCGAAAGATGCCGGCGATTTATGTTTTCAAAATTTGTCT TGTATCCTGGATGCACTAAG AACAGTTGAA ACACTAAACG GTGAGGACGA GAACAACGTCCAATAA

No. KX814424 Cbuq_16395

ATGAATCAGTTAACCGTTGTCGTCTTCTTCGCTTGTGTTGCCGCTTTATTGGCTTCACCTTTGGAACCAAAAGCCGCCGCTGCCCAAGCGAGCCAAGACAGACTGAAAGCCGCTCATCACAAATGCCAGTCCAACCCAGCTACAGCAGTTGAAGAATCTGCCTTGAAAGCTTTAACTAGCGGTGGTCCAAAACCAGCTAACTATGGCGCACATGCGCTCTGCATGTCCAAGGCCCTCGGCTGGCAAAGCGAAGACGGTAGCGTCAACACTGAAACCATCAAAGCTAGAGCCGAGAACATTTTCGGACCATCACCAAAACTGAACGAGATCGTTAATGAATGTGCTCAAAATCAAGCTAATGCCGAAGAAACCGCCGTTCATCTCACTAGATGTTACGTCAAATACGCTCCACGCCACAATGGACATCCCCCA GGTCACCCCC ATTAA

No. KX814425 Cbuq_25979

ATGTCCAGCCTTACAAAAATCGTAGTAATCTTCGCTGTTCTATCCATAACAGCAGCAAAATTTGATGAATCCATGTTATCTGATGATATAAAGACAATTTTGAAAGGCTTACATGACGTTTGCGTCGGGAAGACAGGTGTCGAAGAAGCGCTAATCGACAAATTAAA AGAAGCCGAA TTTTCTGAGGATCAGAAACT AAAATGTTAT ATACAATGTC TTCTAGCCCAAACGGGAGCTATGGACATGGCAGGACATATCGATATTGAAGCGGCCACAGAACTGATACCGGAACAAGTCAAAGCCGCTATGATAAGAGATGTGACACAATGCGCTAAAGAATCAGAACATGTCGCGGAACACTGTGACCGGGCATATACAACTTTAAAATGTTTTTATAAAGTTAATCCTGACGTAAGTTGTATATATGCATTATACGGTTCAATCAAGTATATAACATTGTTAATTTT TAGATCTACT ACGTATTTTAAACAAAAAAA GCGTTAG

No. KX814426 Cbuq_29237

ATGACTGCCGGGGTAAAACTGTTGACCAACAACCGGGAAACCTCCATAGGGAGAAAACGGAATCTGCTGATACCCAGGATAGAAGAAGGGCAATCCACCAGACGGTCTATAAGCCATGATAGCCTGATGCAGTTTTTCGACATCGAGCGGAGCGTTGTTATTTGGGAACCCAGTATTGGCCGTGGGAGTATCAGCCTGTTGGACCTCTTCAGTTGTGGACTGGGGAGCTGGTGTTGTTGGTGGTGTTTCATACGAGGCACACTTGGCACAAGCTGCTTTCATTTCATGGCGAATTTGTCGTACCACGTCTGGTCCGAAACAGCTCTCATAAATCTTCATCATGGCATATTTTTTAATGAATCTGTCTTCA CTGCTGCCAA AAGCCGTTATAACGTTTACA TATACGACTA A

No. KX814427 Cbuq_37516

ATGAAACAGGTTTTGGCTTTGGTACTTTGTGTATCTGTATTGATCATGGGACAGTCTGTGTTAGAGAGATGGGAGAAGCTTCATGATATATGCCAGGCTGATCCAGCTACTTTTGTCGACGAATCCATCTTTAAGAAAATCAAAGATAATGAAACTAACGTCGAACTTCCACCCAACTTTGGCGCTCACGTCTTCTGCATGACAGTGAATCTGAATATCCAGGATCCAAATGGAAAATTCAACAAAGAAGTGACCGCTAAGCTGATAGGAGAGGTCGTTAAAGATCAGGCCAAAGTGAACAAGGTCGTTAACGAATGCGCAGTGAACAAGCCAAATAAAGATGATGCGGCCGTTGCATTCCTTCAATGTCTCG ACAGGAACAA CGTGGACATCGGTCAAAGAG AAACTTATTA G

No. KX814428 Cbuq_46750

ATGAAGGCCCTCCTTGCTATCGTCGCTTTAATCAGCGCTGCTGTTTATGTTACCGTATTTGCGTTTACACTTCTATCGGAAGGAAAAGAGTTTGGCGATGAGGTAGTCAAGCAGTGTATAACGGAAACGTCGATCAGTAAAGATATTCTAGACATGGATACTATCAATGAAGAAAATAGGGATAAAGTTGGATCGTTCGCCTTATGTGTGTCGAAAAAAGTTGGATATCAAGATGATGATGGTAACTTGCAAACCGACGCAATCAAAAAGGCTTTGACATCCTCTGTCGGTAACACTGATCAAGTCAATACCCTGATGCGGAAATGCTTTGTGCAAAAATCACAAGCTAAGGAAACTGCTTTAGCGT CCTTACTATG TTTCAGCGATGAGCTTTCAA GCAATTAA

No. KX814429 Cbuq_47175

ATGAAGACTTTTATCGTTTTGTCCGCTTTTGTATTAGCAGTGGTTTTGGCTGATACAACAAAAAGCTCCTGGAACAGAGTCCACAAAGCCTGCCAAGCGAAACCCGGAGTCTTCGTCGACGATGCTATTTTCGAAAAACTAAAACGTAACGAAAAAGTCACCCTCCCCGCTAACTTTGGCGTCCATGCCCATTGCATGTTGGAAGGCTTCGGCATCCAAAACAGTCAAGGAGCCATCCAGCAAAACGGCATAAAGAAGGCCGTACAAGAAAGCGTATCCGATCCTGC CAAAGCAAACCAGATCGTCAGCGCTTGCTCGGTGAGCAAA AGCTCCAAAGAAGAAACTGC CCTCGGAATT TTCAATTGTT TCGGCAAAAA CAGCGTCGAC ATTGGCCAGTTTTAA

No. KX814430 Cbuq_61968g1

ATGAAGGTACTTTGCTTAGCTTTGTTTTTGCTGGCCGCTATTGTTCTGGGTGATGAGGTACAAGACAGATACG ACAACGTCCACAAAGGATGTCAAAAAGACCCAGCCCTATACGTCGACGATGCCATCTTTGCGAAATTGAAGAGGGGCGAGAAAGTCAATAATCTTCCGGCCAATTTCGGCGCCCACGCCTTCTGTATGCTGAAGAATCTGGATCTGCAAGATAGCCAGGGAAAAATCCAACAGGCCGCCGTTCAAAAAGCTGTAGAGAGATCTGAAGCGGATCAAGTCAAAGCGAAACGAATCACCGCAGAATGTTCAGCGCTGAATAAGGGTACTAAAGAAGATTCCGCTTTGGCATTGTTCGATTGTCTTGGCAAGAA CAGAATTAAC ATCGGTCAACTGTAA

No. KX814431 Cbuq_61968g2

ATGAAGATTTTTGTTGCTTTTAGTGCAGTCCTTTTCGTAGTGCTGGCTGAACACCAACATGAGCATGCACACCAACATCATCCTGAAGACGTGCATGGTTTAGCAAAAGTTCACACAGTCTGTCAAAGCAGCGACAGTACCTATGTCGACAATGACGTCTTCCAGAAACTGGATCGAAATGTACCAG TAGTTTTGCCAGCTAACTTTGGAAAACATT TACTCTGCATGATGAAAGGAATCGGGACAGTGTCTGCTGACGGTCAGCCCAACGTGGAGGGAATTAAAACCCACATCCATCACGTCATTCACGACGAATCGAAAGCAGCACATATTTTAAGGGAATGTGCTGTCGCGAAAAACACACCGGAGCAAACATCGATCGATTTAGAGGCTTGTTTGACGAAACACCACGTTTTCGGTGGTCCCGCTGAACACCATCACCATCCT TAA

No. KX814432 Cbuq_67219

ATGTTAGAAAATGCATCTCAAGAGCTAAGAGATCAAAGAAAAATAATGCAGGAGGAAAGGCAAAAATTAGAGTATAAGAAAAAAAATAGCAAGCAATCTCAAAAAGGTAATAAATCCCAAAGAGTTATGATGTATAATACAAGA CTTTCAAGTAGGGCAGATACAGATTCATCAGTTATTGAAATACAAGATTCTTCAGAATCTAGTAGTAGCGATACTAGTAACTATAATAGTAGCAATAGCGAATTTGATTACGATAGTGACTTTGATCTTTCAGAGAACGTAATGATAGGTCTCCCTGACGCATACTATGGGGGTTATGGAAGGTGTTACTCTTGTGGTGAAAGGGGACATTGGGCAAATG GTTGCCCATT CCGCTAA

No. KX814433 Cbuq_67727g4

ATGTTTAAGGCCAAAAATATTGCGCTGGGTCGACGTCGTATACACATTTCGCTATTTCAAATGCTGCCGTGCACTTATCGTTCAATGTTTTTGCAGCGTCTTTGCACTGTTTCATACACTTTATGCCATGGGGTGCTAGCGATGGTG GTGCCTGTGTTTCCATCATTTTTAAGCCGGCTTCCCAATCAAGAGAATTATCTGGTTTTTGCAACTTATAAAAATTCATTACACATTGTAAATAACACTTTCCTCTTTTATCTATGTCCCAAACACCATCATGCATTCCATTTACTTGTTCTTCAGTTAC TTTGGTTTTT GGAATACAGG CATTTCTAA

No. KX814434 Cbuq_74007

ATGAAGTGGTCTATCGCAATTCTTTTATGCTTTATTTTGTCAACAGTTCTGGCTCTCACAGTCGAAGAAAGCAAAGAAAAGGTCAAGAAAGCCCATGAGAAATGTAATGGTGACGCGGCTACCAAACTTGATCCCGAAGAAAAAAAAGCATACAAAAGCTCCAAAGTAGTAGGACCTAGCTTAAAAGCTCACGCGCTTTGTGTGTCGAAAACGC TTGGCTGGCAACACCCCGACGGAAAAATAGACAAAACATCAGTCAAAGAGAAAATATCCAGTTTCATTACCGATAAGGAACAGGCGGATAAGATTTATAGCGAGTGTTTGGTCGATCACGACGATGAGAAGGACACAGCCCATAATTTGC TTGTTTGTTA TGGAAGACAT TTTGGCCATA AACATTAA

No. KX814435 Cbuq_74056

ATGAAGTATTTTATTTTAATTTCTGTTCTTGTTAGCGTATTTACATGTGGCTTTGCTGCATCGAGAGCTACGTGGACTCAAAAATTCTTTAGTTTTACAAACGAATGTATAGCTGATACCGGTATAGAGGCAGACATTGTCCAAAAGCTTTGCAAGGCCACATTACCAATGATCCTAAGTTGAAGACTTTCCTATTTTGTATGACGAAGAAAGGCGCACTACAAAACGCGAACGGCGAGGTCCAGATTGAAGAGTTCAAAAAACAACTGCCCAGTCTTGTCGAAAATCCTGAAACTACTATTGAGTTGGTTAGGAAATGTGTTTGGAAAGAGGGAACCCCTGAAGATATTGCATTGCAAATATATGG ATGTTTTTACAAAACCGATT CTAATAAATA A

No. KX814436 Cbuq_85742

ATGAAGTTTGTTGTTCTGTTCTGTGTCGTTTTGTTATTGGCAACGGTTGTATCAAGTAAAAAGCATCACAAAAACAACAACGAGGTAACACCAAAAAAAGCTTTTAAGGAGTGTCAAAAAAATGCAACCACCCGTATAGACAAACAAGCTGTTAAGAAATATAAGAAGAAGGAAGTAGACAGTATGCCACAAAATTACGGCGAGCATCTTCTTTGCATTTATAAAGCAACTGGATATATTGGAGAAGATGGTGTCGTAAATCAAGACGTTTTGAAGAAGAAAATAACGAAAAAAGCCCAACCAAGTCAAAACGTTGATACCTTGCTACAGGAATGTGGTGCTGCGAAGGCAGATCCCAAACAGACAGCT ATTAACCTCG ACTCGTGCTT AACAAAGAAC AATCTCTAA

No. KX814437 Cbuq_97345

ATGAAGCTGCTGATATTTGCTGTGTTTTTATTAGTCACTCTATTTCAAGTTAAATGTCAGACCGACAAACAAAAAGAACTCCTCGCGCAGCATTACAAGCAGTGCGTCGAGCAAACTCACGTCGACCAAAATGTCCTGCAACAAGCCCGAGCTGGCAACTTCACCGACGATCCCAAATTAAAAGACCACATTTTATGCATTACCAAGAAGATTGGCTTCCAGGACGAGGCGGGACATTTGCAGAAAGAAGTCATCCAGAAGAAACTGAAAGAGGCGGTGAAAGGCAACGAAGACCAAACCAAAAAACTAATGGAGGCTTGCGCCATCACCAACCAGGACCCTAAACTTCAGGCTTTCAACGCGTTCAAATGCATCCACCACA AAGCTAAGAT CAATTTATTG TAG

No. KX814438 Cbuq_97376

ATGAGTGGTCTACTTGCTTTGTGTGCGATCTTAGCCACGGCTTTGGCCATTACGGTAGCGTATGACTTCGAAG ACGCAGATTTCAATCAGTTTTTAGCGGATGACCTCGAAGACGGGTTAGATACGTTGGATTCTGCGTTTGTTCACTATAGGGTCAGGCGCGCCGAAGATGCCAACCCACCGGCTCAGTCCGGCGATGATAAATGTAAGAAGAGACGTAGGAAACCATCGTTGTGTTGTGCGGATGATATTATCGACCAGCAACATGAGAAAGATCGCGAAACCTTCAGATCTTGCTTCAGGGAAGTGTTGGGTGTTGAGAAGTCCGGTCATCATCGTAGAGGTGACCCATTTAGTTGTAAGGAGGCTGAAAAACGACGTAATGACATGACCTGTGTTACTACATGCTATGGTCAAAAGAAGGGCTTCCTGGATGATCAAGGAAACCCCATACCAGAAGCTCTAACCAAGAGTCTGAAGGACGCCTTCGCTAAAGAAAGTTGGTTCGATGGTGTAGCGGATAAAATTGTGACCACCTGTCTT AAGGAGGCTG ATAATGCCAC ACAATACCAA

CCTAAACCGTCTTCCGATGACATCAAACTATGTAATCCATCAGGTCTCACTCTGAAACACTGCCTGTTCAAGCAGATCCAACTCAGCTGTCCTGCTGACCAGATTAAAGACCAAAAGGCCTGCGACAAGTTTCAAGACAGAATCAAGAAA GGTTTGGATG ATGTGGAACC ACAACCCCCTCCGCCATTTG ATGGTCCCAG AGACGACTGA

No. KX814439 Cbuq_97535

ATGAAATCATTACTGGTTATCTCAACTATTTTGTGCGTGTTCATCTATGTTTTGGCTGATTTGAGCGATGAACAGAAGCAAAAGGTCCTCAACTATGGAAAGGAGTGTATTGCTGAAACTGGCGTGGACAAGGAACTCGTCCTTAAAGCTAGACAAGGGAGTTTCAGCGATGATCCCAAGTTAAAAGCTTTCGCCTTCTGCCTCTCGAAGAAAATTGGTTTGCAAAACGCCAGTGGTGATGTTCAACATGAAGTTTTGAAGGAGAAATTGTCTAGCGTCGTCGATAACGCCGAAACCGTCAACAGTTTGATTTCTGCTTGCGTCCAGAACAAAGGTTCTCCCGAAGAAACGGCGTACCAA ACGTTCGTTT GTTATTATGA GAAAACTCCGTCTCACGCCT CTATATTCTA G
