# Supplementary material for: The developmental transcriptome of the bamboo snout beetle Cyrtotrachelus buqueti and insights into candidate pheromone-binding proteins
Source: PLoS One. 2017 Jun 29;12(6):e0179807. doi: 10.1371/journal.pone.0179807 (PMC5491049; doi:10.1371/journal.pone.0179807)
Supplement: S32 Text — (DOCX) [file pone.0179807.s032.docx]

DponOBP8 [*Dendroctonus ponderosae*],GenBank accession number AGI05175.1

CmonOBP2 [*Cryptolaemus montrouzieri*],GenBank accession number ALW95359.1

TcasOBP13 [*Tribolium castaneum*],GenBank accession number EFA02858.1

TcasOBP16 [*Tribolium castaneum*],GenBank accession number EFA02853.2

TmolOBP5 [*Tenebrio molitor*],GenBank accession number AJM71479.1

TmolOBP6 [*Tenebrio molitor*],GenBank accession number AJM71480.1

TmolOBP8 [*Tenebrio molitor*],GenBank accession number AJM71482.1

RdomOBP2 [*Rhyzopertha dominica*],GenBank accession number AIX97125.1

RdomOBP12 [*Rhyzopertha dominica*],GenBank accession number AIX97135.1

RdomOBP5 [*Rhyzopertha dominica*],GenBank accession number AIX97128.1

HpicOBP2 [*Heptophylla picea*],GenBank accession number BAC07271.1

HparOBP2 [*Holotrichia parallela*],GenBank accession number BAC07273.1

HparOBP [*Holotrichia parallela*],GenBank accession number AEA76516.1

HoblOBP2 [*Holotrichia oblita*],GenBank accession number ACX32049.2

AschPBP2 [*Anomala schonfeldti*],GenBank accession number BAF79600.1

ArufPBP3 [*Anomala rufocuprea*],GenBank accession number BAF91330.1

ArufPBP2 [*Anomala rufocuprea*],GenBank accession number BAF91329.1

AschPBP [*Anomala schonfeldti*],GenBank accession number BAF79603.1

MaltOBP1 [*Monochamus alternatus*],GenBank accession number ABR53888.1

DplaOBP1 [*Delia platura*],GenBank accession number BAS69441.1

DantOBP1 [*Delia antiqua*],GenBank accession number BAI82441.1

CstyOBP [*Calliphora stygia*],GenBank accession number AID61308.1

CstyOBP [*Calliphora stygia*],GenBank accession number AID61313.1

LsatPBP4 [*Liriomyza sativae*],GenBank accession number ALZ41690.1

DwilOBP19d [*Drosophila willistoni*],GenBank accession number XP002064402.2

SfurOBP8 [*Sogatella furcifera*],GenBank accession number AHB59654.1

SfurOBP5 [*Sogatella furcifera*],GenBank accession number AGZ04905.1

LstrOBP6 [*Laodelphax striatella*],GenBank accession number AGZ04925.1

LstrOBP3 [*Laodelphax striatella*],GenBank accession number AEQ19909.1

NlugOBP2 [*Nilaparvata lugens*],GenBank accession number ACI30680.1

LeryOBP3 [*Lipaphis erysimi*],GenBank accession number AJO61166.1

MperOBP3 [*Myzus persicae*],GenBank accession number CAR85644.1

ApisOBP3 [*Acyrthosiphon pisum*],GenBank accession number CAR85630.1

PmegPBP [*Panstrongylus megistus*],GenBank accession number JAC85421.1

CnipOBP4 [*Chrysoperla nipponensis*],GenBank accession number AKW47225.1

CpalOBP7 [*Chrysopa pallens*],GenBank accession number AKM52550.1

CpunOBP3 [*Conogethes punctiferalis*],GenBank accession number AHX37225.1

CmedOBP13 [*Cnaphalocrocis medinalis*],GenBank accession number ALT31643.1

CpunOBP2 [*Conogethes punctiferalis*],GenBank accession number AHX37224.1

SlitOBP28 [*Spodoptera litura*],GenBank accession number ALD65902.1

SinfPBP1 [*Sesamia inferens*],GenBank accession number AEQ30019.1

SnonPBP1 [*Sesamia nonagrioides*],GenBank accession number AAS49922.1

PxylPBP1 [*Plutella xylostella*],GenBank accession number ACI28451.1

AconPBP3 [*Argyresthia conjugella*],GenBank accession number AFD34179.1

MsexPBP [*Manduca sexta*],GenBank accession number AAA29325.1

ApolPBP [*Antheraea polyphemus*],GenBank accession number P20797

BmorPBP [*Bombyx mori*],GenBank accession number P34174

AperPBP1 [*Antheraea pernyi*],GenBank accession number Q17077

AperPBP2 [*Antheraea pernyi*],GenBank accession number Q17078

HvirPBP [*Heliothis virescens*],GenBank accession number Q27388

EposPBP1 [*Epiphyas postvittana*],GenBank accession number Q95VE9

EposPBP2 [*Epiphyas postvittana*],GenBank accession number Q95VF0

SexiPBP [*Synanthedon exitiosa*],GenBank accession number AAF06142.1

RmadPBP [*Rhyparobia maderae*],GenBank accession number AAM77027.1

LoryOBP [*Lissorhoptrus oryzophilus*],GenBank accession number AHE13799.1

DponOBP8: [*Dendroctonus ponderosae*],GenBank accession number AGI05175.1

CmonOBP2: [*Cryptolaemus montrouzieri*],GenBank accession number ALW95359.1

TcasOBP13: [*Tribolium castaneum*],GenBank accession number EFA02858.1

TcasOBP16: [*Tribolium castaneum*],GenBank accession number EFA02853.2

TmolOBP5: [*Tenebrio molitor*],GenBank accession number AJM71479.1

TmolOBP6: [*Tenebrio molitor*],GenBank accession number AJM71480.1

TmolOBP8: [*Tenebrio molitor*],GenBank accession number AJM71482.1

RdomOBP2: [*Rhyzopertha dominica*],GenBank accession number AIX97125.1

RdomOBP12: [*Rhyzopertha dominica*],GenBank accession number AIX97135.1

RdomOBP5: [*Rhyzopertha dominica*],GenBank accession number AIX97128.1

HpicOBP2: [*Heptophylla picea*],GenBank accession number BAC07271.1

HparOBP2: [*Holotrichia parallela*],GenBank accession number BAC07273.1

HparOBP: [*Holotrichia parallela*],GenBank accession number AEA76516.1

HoblOBP2: [*Holotrichia oblita*],GenBank accession number ACX32049.2

AschPBP2: [*Anomala schonfeldti*],GenBank accession number BAF79600.1

ArufPBP3: [*Anomala rufocuprea*],GenBank accession number BAF91330.1

ArufPBP2: [*Anomala rufocuprea*],GenBank accession number BAF91329.1

AschPBP: [*Anomala schonfeldti*],GenBank accession number BAF79603.1

MaltOBP1: [*Monochamus alternatus*],GenBank accession number ABR53888.1

DplaOBP1: [*Delia platura*],GenBank accession number BAS69441.1

DantOBP1: [*Delia antiqua*],GenBank accession number BAI82441.1

CstyOBP: [*Calliphora stygia*],GenBank accession number AID61308.1

CstyOBP: [*Calliphora stygia*],GenBank accession number AID61313.1

LsatPBP4: [*Liriomyza sativae*],GenBank accession number ALZ41690.1

SfurOBP8: [*Sogatella furcifera*],GenBank accession number AHB59654.1

SfurOBP5: [*Sogatella furcifera*],GenBank accession number AGZ04905.1

LstrOBP6: [*Laodelphax striatella*],GenBank accession number AGZ04925.1

LstrOBP3: [*Laodelphax striatella*],GenBank accession number AEQ19909.1

NlugOBP2: [*Nilaparvata lugens*],GenBank accession number ACI30680.1

LeryOBP3: [*Lipaphis erysimi*],GenBank accession number AJO61166.1

MperOBP3: [*Myzus persicae*],GenBank accession number CAR85644.1

ApisOBP3: [*Acyrthosiphon pisum*],GenBank accession number CAR85630.1

PmegPBP: [*Panstrongylus megistus*],GenBank accession number JAC85421.1

CnipOBP4: [*Chrysoperla nipponensis*],GenBank accession number AKW47225.1

CpalOBP7: [*Chrysopa pallens*],GenBank accession number AKM52550.1

CpunOBP3: [*Conogethes punctiferalis*],GenBank accession number AHX37225.1

CmedOBP13: [*Cnaphalocrocis medinalis*],GenBank accession number ALT31643.1

CpunOBP2: [*Conogethes punctiferalis*],GenBank accession number AHX37224.1

SlitOBP28: [*Spodoptera litura*],GenBank accession number ALD65902.1

SinfPBP1: [*Sesamia inferens*],GenBank accession number AEQ30019.1

SnonPBP1: [*Sesamia nonagrioides*],GenBank accession number AAS49922.1

PxylPBP1: [*Plutella xylostella*],GenBank accession number ACI28451.1

AconPBP3: [*Argyresthia conjugella*],GenBank accession number AFD34179.1

MsexPBP: [*Manduca sexta*],GenBank accession number AAA29325.1

ApolPBP: [*Antheraea polyphemus*],GenBank accession number P20797

BmorPBP: [*Bombyx mori*],GenBank accession number P34174

AperPBP1: [*Antheraea pernyi*],GenBank accession number Q17077

AperPBP2: [*Antheraea pernyi*],GenBank accession number Q17078

HvirPBP: [*Heliothis virescens*],GenBank accession number Q27388

EposPBP1: [*Epiphyas postvittana*],GenBank accession number Q95VE9

EposPBP2: [*Epiphyas postvittana*],GenBank accession number Q95VF0

SexiPBP: [*Synanthedon exitiosa*],GenBank accession number AAF06142.1

RmadPBP: [*Rhyparobia maderae*],GenBank accession number AAM77027.1

LoryOBP: [*Lissorhoptrus oryzophilus*],GenBank accession number AHE13799.1
