# Supplementary material for: The developmental transcriptome of the bamboo snout beetle Cyrtotrachelus buqueti and insights into candidate pheromone-binding proteins
Source: PLoS One. 2017 Jun 29;12(6):e0179807. doi: 10.1371/journal.pone.0179807 (PMC5491049; doi:10.1371/journal.pone.0179807)
Supplement: S2 Table — (DOCX) [file pone.0179807.s034.docx]

| >*Cbuq12614_g1* | | | |  | |  | |
| --- | --- | --- | --- | --- | --- | --- | --- |
| AAATTGTTTTTATTCATTTTTTTTTATTTAAAAAAAGTACCCGTAAATGAATCACCCTGT | | | | | | | |
| ATTATATATATAATAAAGTTTCATTAAAACACAGTATCCAGAAAATAATCAGGAATAAAA | | | | | | | |
| AATATTATTTATTTAAAAAAAATCTACACAAGGGTATTTACGTTGTAATAATCATAAATA | | | | | | | |
| AATGAAATATTACAAAATGTCTACGAAGGTTTATTAAACCTTTCAACGTCGGATGCTCCC | | | | | | | |
| AACTCATCTGTTAATCGAGATTAACACATTATCTATTAAAATGACTGTGGAAGAAAATTA | | | | | | | |
| TTTTACCCAGCACATCTGCTTTCAGAGAAAGTAGGTCATATCAACTTGATAGTCTATAAA | | | | | | | |
| AAACAATAATTGGTGATCTTAGAAAAAGAAGTGTTTAATTAAGTCTAAGCTATTATAAAA | | | | | | | |
| TCACAATATTACAAGTATCAGTAAACAACTGAACAACGGTAGTTATTTAAGCTTTCTTCT | | | | | | | |
| CCTCCTTTTTCGGTTCGTCTTTTTTGAACAATTCCGGATGCGTGTGTTCGAAATAGCACT | | | | | | | |
| TGTGTATCAAATAAACGGTATCGCACGCGTCCTTTCCTTTAATTTTACATTTCTCCACGA | | | | | | | |
| TTGCGAGGGCCTGTTCTTTATCGGCCTGCGCCGGCAGTTTGGCCTTTACGACGTCCAACT | | | | | | | |
| GTACTTCGCCCTCGTCATTCACGAATCCCGCCTTCTGGTAGAAACACTTGGTGAAACATT | | | | | | | |
| TGAGGGAGGTGTCTTCCGCGAAGTTTCCTTGGTCCGCTTTCTCGATCAGTTCCGGATTGA | | | | | | | |
| CTTTGCTCTCCTCAATGCATTGTTTACGGTTCTCGAGGATTTTCTTCTTTTGCTCTTCGG | | | | | | | |
| TGAATTCCTGGCATGAAATGGCTGACACTACGATGATCAACACTGACACTTTCAATAGTC | | | | | | | |
| CGTTCATGTTTGGTCCGCTCGCAAGCTGCAATTAAACCCACTCTACCAAAAACCAATTGT | | | | | | | |
| TTATATATACGTGTCGGGTGCAGCGAGGTCGCGAAATTTACTGTATCTCCTCCTGTACCA | | | | | | | |
| GTTTGTTCGCTTAAATCGACCATTTGGGGGGACAAAATCTATTGTATTTCATATGGAGTT | | | | | | | |
| TATGGGTTTTACGCCGGCGATCTCCATTATCGATTCGACGCGACCCAAACAGATGGAAGC | | | | | | | |
| CACGAAGTTCG |  |  |  |  | |  | |
|  |  |  |  |  | |  | |
| >*Cbuq16395_g1* | | |  | |  | |  |
| GGGGCAGGTATTTATTTCAGATATTGGGCGAATTGTATTTAAGTATCAATTCAACTTAAT | | | | | | |  |
| GGGGGTGACCTGGGGGATGTCCATTGTGGCGTGGAGCGTATTTGACGTAACATCTAGTGA | | | | | | |  |
| GATGAACGGCGGTTTCTTCGGCATTAGCTTGATTTTGAGCACATTCATTAACGATCTCGT | | | | | | |  |
| TCAGTTTTGGTGATGGTCCGAAAATGTTCTCGGCTCTAGCTTTGATGGTTTCAGTGTTGA | | | | | | |  |
| CGCTACCGTCTTCGCTTTGCCAGCCGAGGGCCTTGGACATGCAGAGCGCATGTGCGCCAT | | | | | | |  |
| AGTTAGCTGGTTTTGGACCACCGCTAGTTAAAGCTTTCAAGGCAGATTCTTCAACTGCTG | | | | | | |  |
| TAGCTGGGTTGGACTGGCATTTGTGATGAGCGGCTTTCAGTCTGTCTTGGCTCGCTTGGG | | | | | | |  |
| CAGCGGCGGCTTTTGGTTCCAAAGGTGAAGCCAATAAAGCGGCAACACAAGCGAAGAAGA | | | | | | |  |
| CGACAACGGTTAACTGATTCATTGTCTTGTTTGAGTAGTTTTCGGATACACTAATCGGAA | | | | | | |  |
| CACTAATGTCATTGGAACGACGTTACTCTTATTTATATGACGAAGTGGTCAAAGATTTTC | | | | | | |  |
| CTTTATCATGCGTAAATACTCTTTTAACAACCTAATTATTTTGAATGTTAAGAATGTTAA | | | | | | |  |
| GGGTCAGATATTATCGGTCTTCTAATCAACGTTTATGTTGATAAAAAATCTTTCTTCTTT | | | | | | |  |
| TTATTTTTAGAACGCTTCCTTTATTATAAAAAATTAACGATATCGAATCATTTATTACGA | | | | | | |  |
| TTTTCTTGCATTCAAAATTT |  |  |  | |  | |  |

| >*Cbuq25979_g1* | |  |  |
| --- | --- | --- | --- |
| GAAGAAACAACAGACGAGCGCGTGAACCAAAACCGCTTGGCAGGAATCGTTGAAAAACAA | | | |
| AAATTATTGAAAATCGAACCGTTGGTACCCCCTAACGATCGGAGCTGACCAGCGGCAGTG | | | |
| GGTGTCCTATAAGAACCGAGTCAGCACTCTCCTAAAGACTTTCTTTAAAATCGACCGATT | | | |
| CGAAGTGGTGCAATAAACAAATGCGTGCCGTCGATACAGTGTGCTTTGGTGATGTTCATC | | | |
| GGTTCATGAGATAATAGTGACGTGTGACATACAATTGACATTTTATTTTATAATTATTTT | | | |
| GGATTACTATAACAATGATTCCTCGAGAATACAAGCGGCAGCTATTCCTCACCTATACGC | | | |
| TTCTCCTCATAGGAAAAGTACATTCTCAAGGTGGACGGACCTACGATACAAGCGACTGGA | | | |
| TACCTGTAACATCAGCACCTCTCGAAGGTCAGGCTGACAACCACCAAGAAACTCCCAACG | | | |
| ACAGAATACTAAGCCTTGAAGTGCCGACTCAGCAACAGTTTCAACTTACAAACAAAGAGT | | | |
| ACCAGAACGTCCAGCAGGAACCTCCGGCGAAATCACAGCAAATCGTCAGCCCCACATCCC | | | |
| GGAAGCTGAAGTACCCGTTAGAGTACCAATTCGAGGCGCCGTCTGCATCACTAAAACAAA | | | |
| ATCCTAGTGAAAATTATTATAACGGTGGGGGTAGTGGTGATGCTCCTTACGCTTTTGAGG | | | |
| TTCCCCCAAAACCCTCGCCAGCGTCTCAGTTAGCTCAGCAACAACAACAAAACTACTTTA | | | |
| AACCGGCTCAAAAAGCTCCATTAGAGAACTATGTACCTACTCTTAATGTAAATCCAGAGG | | | |
| TACCACCCGTCAACGCGCCAAGTACTTACGAAAAGTACCAACCTCAAATTAACGCCAGTC | | | |
| TCTTTAAATCAGTAAATCCGACAACTATTAACGATGGGTTGTACCAAGATACTAGCAGTA | | | |
| AAGGTCAACAAACAGCAGTGCCAGCACAAAATCCTCCATCCACCGTGATCCATAAGACAT | | | |
| TACCAATCTTAGAGAAACATTACACCACTCAAAGTAACGATGAGCCTAAGCAAAACGTTC | | | |
| AACTTGTGTATGTACCTGTCGAAAACCTAAAACCTACCCAACCGGTGAATCATCAGTTCA | | | |
| AACCTAGAACCCCGCCAACACTCGAAACACCAAGATCGACCGTACCAACGCTCCATTCAT | | | |
| TCAAACCATCGTTGCCAGTACCCACCCGACCACCCCCACAGTTTGGATTCATCAATTCAC | | | |
| CGAAACCAAGCAGTTTCAATTTCAACCCCCCAGCACCACCACCACCACCAAGCGATAACA | | | |
| AAAAGGAGAAACTGAATAACATCGAGAAAGACTTCATTCAACAGGCGTTGTACGCCCACA | | | |
| AGCTTCAGAAAACCCTTCAGGACGAGATCAATCCCGTGAACCAAGAAAACGGTTCCAAGA | | | |
| GACGCAGACCACACCAACCAGCACTCGCTGTGTTCTTACAGTCGGAAAAAGAAGCGGAGG | | | |
| TTAACGATGTGCTGCACGTGCTCAAAGACGCCAAGACTATATCTGTACAAGACACGTTAA | | | |
| CTCGAAGTTCTCCGAATATATTTATCGGACCTTCCACCTTGGAATCTCCACTAGGATACA | | | |
| CGAAATTCGCCCTGCCGTATCTGAATAATCTCAACGGAAACAGAATAGAGCGGAAAATCG | | | |
| ATCAGTTGCCGTTCTTCGTGGCACCGGCGAGTTATACCACTCCTGACGGTTACTCCAAGA | | | |
| TTCCTCTGCCATCGCCTCACGTCGGATCCATCGTCGTATCTTTACCGAAGAGTCCAGCTC | | | |
| GAAGCACGGTCTCCCCGCCAGCTTACTTCAACCCGAACGATTTCGGTTTAAGTTTTCCGG | | | |
| CTTATAGTCAACAGAACTACATCAATCCGACTTTCCAGCCGTTTCAGACGGACGTAGGGT | | | |
| CCTATCAAAACAATCCGGCGCCGTCGCAATCGTTCACGCCGCCCATTCCCGATGTCCAGG | | | |
| ACGCTTACAACGTCAGACAGCCTCAGGAAGTGTACTCGACAGACTTGGGGCAACGTGGGT | | | |
| CCAATCAGTATAGACCCACTGAGGAGAATTACCGAACAACCACCCCAAGAGGGGAGATCA | | | |
| TCAACGAGGACAAGTCACCTCTGGGTAATGTAAACTCGTTGGACTTGGCTATCAATAATT | | | |
| ATGAATTAAAACATGTCAACAACCAATTTGTTGATAGTAATAAAGGGAAAGCTACCGGTA | | | |
| GCAGAAAACCACAACATGCCCCATCAAACGAGTACACAACGACAACAAATCAACCATTTG | | | |
| TGCAAGATACCTACCAATACACCACCGAAAAAGTAAGCAGAGGTAGAACGAGAGGCAGAA | | | |
| GCCGGGTAACATCTAAGGGCCCGAGCAAACCAGCTCTAGATGACTATAAATTCACGGTGT | | | |
| TGGAGGACTTTTTGTCTACTATGAACAAACCAACTGAAAGCTACTACTCTCAGGACGATT | | | |
| TTTTCTCAAGCACGTCATCAAAACAGGATGACTTCCACCAAGGTCTGCCTAAAAACACAA | | | |
| GAAAAGAAACCGATAACAATAATTTCTACCAGAGCAGCGGGAATATCTACAGTCTAGCCC | | | |
| AGCCGCAGCTTTACGATAACACCGAATACGAAAAGACCAAATCAGGCACTGTCGATGCTG | | | |
| CACCAGAAATTGAACCTGACATTCCTGTCCAAACGCCCTCACCTTTCAATTACTCGCCCG | | | |
| GTGGATTTGAAAACCCAGATCCCCCAGTTGAATACCATCACCCAACGTATCCACCAGAAC | | | |
| GTCAACCGGAAATTGCCGAAGTTACAACTCTACCTCCACCAGCTCCAACAAAAAGACGTA | | | |
| GGTTAATGAAGAAGAAGCCTGAGAACACTGAAATCGATACAGAACCACCAATATCATCAC | | | |
| TAGGTTTCGAACAAGTACCATCGCCCAGCTTTGAACAAACACCCAATCAATTTCCAGTTC | | | |
| AGCCAGACCCCGTTCCGCAAACTTATAACACAGCACCCGCCGCTGATGATCCTCTGAACC | | | |
| AAGATGACGTCCATCGTCTCAAGCAACTGGCTCCTGATTTGTTCCAGCCGAACTTTGATC | | | |
| AGACTTATATCAGCCTGCAGGACCAGGCAGTTAGGTCTCTGTTGACGCCCAATATCCTGC | | | |
| CACTTACAACGCCGGAAGAATCACCGAAACCGTTCGTGTCGACTACTGAGTTTCCCGTTG | | | |
| AAATAGCTCCCAGCACTGAAACTGAAAAAACTACAACTACTAGGGGCAGAGGAAGAGGAC | | | |
| GAGGAAGAAGTCGTGTTTCAACGACAACTTACGCCAGTGCCGAAAGGCATGCTGTTACCA | | | |
| ACGCCCCCAGGAGGACGTCAAGTCGACGAAGACCTGTATCAAGAACAACAACAGAGAGAG | | | |
| TGCCCTCTAGTACTGAATACGAGTATCGAGAGGATTCTCCCCGAACAAATTCGAGACAAA | | | |
| GAACTAGATCCAGAGGCAGACCAAGTTATCATCAAACAACTACAACCACCACGACAACCG | | | |
| AAGCAATTCAAGTTATAAAAGAACAGCCACTGGAGGAGATATATCCCACCGCATACAGTG | | | |
| AACAATTAAAGCTGGAGAGCGTTCAACAATACCAGCCACTGGAGGAACAACATTTATCCC | | | |
| CGACTGAAGCAAACTACCTCTCAAATCCTTATGCGGCACCAGCTCAAAACACAGATTACG | | | |
| ACCAATACAATTACAACCCAACGTTAGCACCTGCTCCAGTACCAACCAATGTTTATCCCT | | | |
| CCGAAACCCATCCACCTGCAGTGGTTCACGAGATCAACACTAGTCCTCCGGAGATTGAAC | | | |
| AGCCGGCTCGATATAACAACGCAAACATCCGATCTGGGGGAATCCTCAACAACAATCCGG | | | |
| AAACAACCACCAAATCGGCACCGAAAATCAGAGGACGTACAAGGGGTAGGTCTCGATTTG | | | |
| CCATTTCAACCACGAGTACAACAAAACCACGACCTGTAACCAGATCTGCCGTGACCACAC | | | |
| CACCAGAACCCGTGGAAAACGTAGAGGAAGAATTTTATGGTTTCATTAAACCTCCCAGTT | | | |
| ACCAACAGAATGACGTTCAACAACAATATCTCACTCCCATCCTACAAGAAGAGACCAATG | | | |
| ATGAGCCAACTATACAATTCGTTGGAGAAATCCGACCGAAATATACCTCAGCCCCTTCGG | | | |
| CACCGGAAATCGTAGAAGAACAGATAACAACGACAACTACAATCACCACATCAACGACAA | | | |
| CCGAAGCCCCTAGGTCTCGGGTGAGATCAAGAACGAGAGGATCATCGAGAATAACCTCCA | | | |
| ATAACAGCTATAAACATACTGATAATGAAGTGAACGATTCGAAAGTTAGTGAGCAATCAA | | | |
| CGAGAAGACCTATCCGAACTAGGGGAAGAGGCTCGTCCCATTATCGAGCCCCCGAAAACG | | | |
| CAAAACGAGGAAATAACAATGAAGATGTCGCGAATCAAAACTATCCCGTTAATTTCTTGC | | | |
| AAAAATTCGAGACAAGTACCACTCCAAAACCCACCGTAGCCAGTTTACAAATAACCATAG | | | |
| ATCCCTCGGAGGAAGATGTCGCGGAACCGGAAGATCAGTTCTCGTTTTCGTCGTTCTATT | | | |
| CAGCTAAAATAGTTCCTGCTCCAAACCTGTTAAAGCAGGACGAAAAAAAAGGCACTCGGA | | | |
| AAGAGGAGAAGTCTCATGACCTTCCCGAGCTGGTAACCGAACCATTAAATGTTAAGCCAG | | | |
| AAACAAGTGAGCTGAAAGAACCTGAACCGGAAGCTTTACCTATGATCGGCGTCACAGAGA | | | |
| ACGTTAATAGCTCCAATGACGAGGAGATAGATCGTCAAAGCTCCAACGAAGGAAACTTAA | | | |
| CAACTGAAAAATACCTTTCTGGAGGGAAATCCAAGGGTAAGCGAAGAGGCGTGTGGAAAC | | | |
| TCGTTAGAACGCAGCCAGTAGATCCCTTAGACGTCTCTGAATCTCAACAATACGAAACAG | | | |
| TTATAAACGATTTCCAAGAAATCGCTAAAGTTGATCCATTAAACAAGAAACTGTACACAG | | | |
| CCAAACAATCATCACCGCAAAAAACGGCTTATAATAAAGCCAACTTTGTTAATGAATTTG | | | |
| ACGTAAATAATCTAGATGATGATGACGTCACAACTGCGCGAGTCATACCCGATAATGACG | | | |
| AGACGAGATTCGCCGAAGAAGAACCAGATCCATCAACAAAACCAACATTCAAATTACAAG | | | |
| AAAGTATTTTCGAAACAATCTACGAAATGTTCGGTATCACGGAGAAATCCACTAAAATTA | | | |
| ATAACACCACCACAACAACACCAAGCCCTACAACAACACCAATTAATCCCACCTTTCCCG | | | |
| ATGAAATAACAGAAGAAATTGTTAGTAGCACAATATTAAACTACGAAGATCGCAATGACA | | | |
| CCGAAGAAACCGATAATACCGACGGATATAACGAAAAGGACGAAGAGGACAGCGCCAGTA | | | |
| CGGAGGACATCGGAACTACTTCGACAACCACCGGAAAATATGACGTTGAGCCGTGGAAAA | | | |
| TGAAGATTATAAAAACGTCTACTTCCACTGAGATATCTCACGAAACTGAAATATGTTACA | | | |
| AAGGTCGATGTGTCAAATCTAAAGGGAAACCAACGACGACTAAGTAGACGTATCTTCTTG | | | |
| TAGACTTAAACAAACAGCTGATGTATATATAAACGTGACTGTTGTGTGGGTGATAATACT | | | |
| TAGTATATCTAGTTAATAAAAACGACATATATTATAAATGCACAATTTCAAAATAAAAAC | | | |
| AAAGTACCTATACGATATGTAAGAACCAAGATATTTCTCGCAATATAAATGTATGCATGT | | | |
| GTAGAATGTGAACTAAATTATTTCATGGTTTCGTAATAAGATTACTAGCTGAATTTTAGA | | | |
| TGTCTCGCAATTAAGATATTATATTTTTTATTTATTGTTTATACATTAAAATTGAATAAA | | | |
| AGGTTTTAATATAGTGTTGTAACTTAATAATTCCCCAAAAATATATTTTATTGTCTCAAA | | | |
| ATAAATAACACATGTCAATAATCATCTGTCACCTGTCAATGAAAATAGAACAATTTTAAC | | | |
| ACACTGTATAAAACGAATTCTTTACAAAAATATATATTTTTCGATGTTCTGCATATTAAA | | | |
| CAATTATTAAATAATTAACAGGTGATACCAATACTAATTACCTGTAATTTATTTTTGAAA | | | |
| AAGTGATATATAAAAGCGGGCAAAATCCTAAAATTAAATATTAAGCCTTAAAAATGTCCA | | | |
| GCCTTACAAAAATCGTAGTAATCTTCGCTGTTCTATCCATAACAGCAGCAAAATTTGATG | | | |
| AATCCATGTTATCTGATGATATAAAGACAATTTTGAAAGGCTTACATGACGTTTGCGTCG | | | |
| GGAAGACAGGTGTCGAAGAAGCGCTAATCGACAAATTAAAAGAAGCCGAATTTTCTGAGG | | | |
| ATCAGAAACTAAAATGTTATATACAATGTCTTCTAGCCCAAACGGGAGCTATGGACATGG | | | |
| CAGGACATATCGATATTGAAGCGGCCACAGAACTGATACCGGAACAAGTCAAAGCCGCTA | | | |
| TGATAAGAGATGTGACACAATGCGCTAAAGAATCAGAACATGTCGCGGAACACTGTGACC | | | |
| GGGCATATACAACTTTAAAATGTTTTTATAAAGTTAATCCTGACGTAAGTTGTATATATG | | | |
| CATTATACGGTTCAATCAAGTATATAACATTGTTAATTTTTAGATCTACTACGTATTTTA | | | |
| AACAAAAAAAGCGTTAGTAAAATAATTTTAGAATAGGACGTAGTTAAATATTCATGTAGT | | | |
| GAAATATATTTCAATGTTCTTCAAGCAATAATGTGTTATTTTTCTATATGAGTGGTTTGA | | | |
| TCCAGTCGACCACTTTAGTATCATCCCAAACATATTTTCCCTTAAATGTAGCTTCTAGAG | | | |
| CCAAAAATAAGGGGGCTCTGGCGCCCTGTTCTGGTGTTAACACTCCTCTGTGACTCGTCA | | | |
| TGTCAGTATCAACGTATCCAGGATGAACAGCATTAACAGCAATATTTCTGTTTGGAGTTT | | | |
| CAGCATCGAAAAGCCTTTGTTGGACGAACGTCAACGCACTAACTCCAACTTTCGATACAG | | | |
| CATAAGCTGACGAGCCCCATCCTTCTTCTACGTTCTTACCTGCTTCGGAATCTCTTACAA | | | |
| ATTTTTCCATCAGTTGATTCAATTCTGCAATAGTTAACTTATCGCTCTTCAATTGTTCTT | | | |
| GTAAAGTTTGCGATGGAATTTTAGATAGATGACCCATCATACTCGATACATTTACTACTC | | | |
| GAGCATTTTGTCGAAGAAGTGGAAATAAGGCTTCGCAAATTCTCAAAGTGGCGAAGTAGT | | | |
| TGACACGAATTGTTTCTTTCGCTTGTGTACCGAAAGACTCTTCGGAATTGCCCGGGAAGG | | | |
| CTATTGCAGCATTATTTACTAAAACATCAATGCCTCCATGCTTTTGTTTCAGGTATTCTT | | | |
| TAAAAGCATCGACGCTGTTCTGGTCAGTAATGTCAAGTTGGTGAAACAGGGGTTTATAAC | | | |
| CTAATTTTTTTAGAGCCTCCACTGCGGCTTCTCCTCTTTGTACATTTCGCGCTGTTAGAT | | | |
| AAACTTGGCCATCGTACTTTTCACATAATCCCTTAACAATGGCAAAACCTATGCCCTTAT | | | |
| TACTTCCTGTCACTACAGCAACCTTAGGAGACGCCATTTTAGTGAACAACTAAATCAAGA | | | |
| TCCATTACCTAGTACTTTCTTATATCTCAAGGTGACAAAAATAAATTATTTATCAAAGTA | | | |
| AGCGGGTTGCCGTAAAAGATAAAAGCTATCTTTGG |  |  |  |

| >*Cbuq29237_g1* | | |  |
| --- | --- | --- | --- |
| TTTTTTTTTTTTTTTTTTTTTGGTGGATTAATGGTTTTATTAAAATATTAGTCGAATTTT | | | |
| GACAATGAAATTATGTCTTTGAGGACAGACTACTCCATTGTGAAAATCCAAGATCAATAA | | | |
| ATAAAATGAGCGCATAATAAATAAAAGGTCAAGTCAATGTAAGGCTACAGCAGTCAATTT | | | |
| TGTATACATTAATTTTTCTGAGAAATTGGTTATATAATAGCCAACATATAAATTAGATAT | | | |
| GTGTTCTATCCGGCAAGAACGTCTCGGTTTATTCTAAATCTATAGGATACCGTCAACATC | | | |
| GAATCCACTATCACTAGTATACAAGAAGTCGTACATCGATGACGCTAGATCATCAATCTC | | | |
| CTTCTCTTTGTCATCGATTTTCCCAGCTTTCCCCTGTCGGCCGGTTCTTCTCAATTCAAT | | | |
| GTCACCGTTTTCAAAATCGTCAGAAACTCCGGCGTATTTTTCTCGAACATCCTTCATGAC | | | |
| GCAGGCTTCTAGTTTCTTGTGCTTGTAACATTTGAAGAAGAACATTGGTCGGATGAGTTC | | | |
| CCTCGATAATGGAGATTTATCCTTGTTAACCTCTGGAACGCATTGTGAGAACTGTTTGCA | | | |
| GAAAGATACACCATCTTGCATGTCTCTCTTCAGTTCATCATCCACGGGCAAGTTACCTAT | | | |
| TCTTTCGCTGATTTTGCTGAAGTTGGGTTCCAAATTATCATCTAGATAACCTAATTCTTG | | | |
| CATAACACAGGTAACGTTCTTTACTTTCCCGCTCATTCTGGACGTGATGGCTTCGATCTG | | | |
| GGCTCTTACATCCATATCTCTCGACATCCGATGACTGCCGGGGTAAAACTGTTGACCAAC | | | |
| AACCGGGAAACCTCCATAGGGAGAAAACGGAATCTGCTGATACCCAGGATAGAAGAAGGG | | | |
| CAATCCACCAGACGGTCTATAAGCCATGATAGCCTGATGCAGTTTTTCGACATCGAGCGG | | | |
| AGCGTTGTTATTTGGGAACCCAGTATTGGCCGTGGGAGTATCAGCCTGTTGGACCTCTTC | | | |
| AGTTGTGGACTGGGGAGCTGGTGTTGTTGGTGGTGTTTCATACGAGGCACACTTGGCACA | | | |
| AGCTGCTTTCATTTCATGGCGAATTTGTCGTACCACGTCTGGTCCGAAACAGCTCTCATA | | | |
| AATCTTCATCATGGCATATTTTTTAATGAATCTGTCTTCACTGCTGCCAAAAGCCGTTAT | | | |
| AACGTTTACATATACGACTAACACCAGAAGACCTAGATTATATTTCATTTTGATTTGATA | | | |
| TGATGGTCAGCTAATGATCTTATCTGGCGTCACTGATCGGCAAGGACTCGAGCGCGTCTA | | | |
| TTTCAAGGACAAGGTGCGGACGATGGAAGCGTCAAACGACGGCACACGGAAAAGCCAAAC | | | |
| TTCTTCGGCGGCCTTCAGGTGTCGTTCGTTATATACCTTCCACCTCTTCGTGTTGTGATA | | | |
| ACCTCGCTTCGTGTCAAGTTCAAGTGTAACCGTACACCGGTTGAGGTTCGGAAAATGTCG | | | |
| CCATATCTGATCTGGTTTCTATAAATAAGGTCG |  |  |  |

| >*Cbuq37516_g1* |  |  |
| --- | --- | --- |
| GGCGATTTTAAGTAATAGTATATTATTAATGTAGCAAATATAAAAGCAAATTAAAAAAAA | | |
| ACAATACTTTGCTAATATCTATGTATTAAATCTTCACAAAAGAAAGATAGATCATATAAC | | |
| AATACATTCTGAATCCTAATAAGTTTCTCTTTGACCGATGTCCACGTTGTTCCTGTCGAG | | |
| ACATTGAAGGAATGCAACGGCCGCATCATCTTTATTTGGCTTGTTCACTGCGCATTCGTT | | |
| AACGACCTTGTTCACTTTGGCCTGATCTTTAACGACCTCTCCTATCAGCTTAGCGGTCAC | | |
| TTCTTTGTTGAATTTTCCATTTGGATCCTGGATATTCAGATTCACTGTCATGCAGAAGAC | | |
| GTGAGCGCCAAAGTTGGGTGGAAGTTCGACGTTAGTTTCATTATCTTTGATTTTCTTAAA | | |
| GATGGATTCGTCGACAAAAGTAGCTGGATCAGCCTGGCATATATCATGAAGCTTCTCCCA | | |
| TCTCTCTAACACAGACTGTCCCATGATCAATACAGATACACAAAGTACCAAAGCCAAAAC | | |
| CTGTTTCATCTTGATAATTGGTAATTAACAACTGAA |  |  |

| >*Cbuq67219_g1* |
| --- |
| CGAGAGCCAATCACGATGCACTCATAATCGGAACCCGGGCCGTCTCAAAAATATAGGTTA |
| ACCAGTTATCAAAATTACCTTATTATTTTGTTAGGTTATTAAAGATATAAAATGACTTGT |
| TTTGAAAATTAATTTGTCAGATTATTTTATTTTGTTGAGTACAAAATTCAGAATGTTTTG |
| CATAATTTCAAAGCAAACCCTTTTATCAAACTTATTTACTAAATTAAATCATAAATTATT |
| ATTAGACGTTTCACAAAACGTTTTACTAATAAAAAGTTACCACACAGTTAAAAGAAAAAC |
| GGCGAATGATGAAAATTTTTTATCTAAACATTTATATAACAAAATTAAAGCCAAAGGGCC |
| TATAACTGTAGCTGATTATATGAAAGATGTTCTTCAGAACCCTACAAAAGGATACTATAT |
| GTCCAAAGATATGTTTGGCGAAAATGGGGATTTTATTACATCTCCAGAAATATCACAGAT |
| TTTTGGGGAAATGTTAGCAGTATGGTTTTTAAATGAATGGTCAAAAATTGGTTGTCCAAA |
| ACCTTTACAAATTGTTGAATTGGGACCAGGACGAGGTACTTTAAGTAGTGACATGTTAAG |
| AGTATTTAGCCATTTTAAAGCACTTCATCAAGTATCTCTACATTTAGTTGAGGTTAGTCC |
| AGTTTTAAGTGAAATTCAAGGAAAAAAATTGTGTTCACAAAGCTATTTGACCAATAACTA |
| TAATAGTCCTATCTATAGAAGAGGTATATCACATGAAGGTATACAAGTACATTGGTATAA |
| ACAATTTAAAGATGTTCCAGATGGTTTTAGTCTTATATTAGCGCATGAATTTTTTGATGC |
| ATTACCAATTCACAAATTTCATAAAACTTATGCTGGGTATAAAGAAGTTCTTATTGACAT |
| AGATAAAATTTGTAATAATGATGAAATCAAATTTAGATATGTCCTTGCTCGTCAAGATAC |
| TCCCATGTTAAAGGTGTTGCTAAAACCAAATGAATCTAGGGATCATGTGGAAATATCACC |
| AGATAGTATGCTTATATTAGAACAAATATGTAACAAAGTAGTGGGTAATGGAGGCATAGC |
| ACTTATTTGTGATTATGGACATAATGGAACTGGAACTGACACATTTAGAGCCTTTAAAAA |
| ACACAAACAAGTAGATCCCCTAATACAACCTGGTACTGCTGACCTTACAGCAGATGTAGA |
| TTTTAATATAATAAGAGAGATAGCAAAAAATAATGGCGATGTTATTTACTTTGGACCTAT |
| TAAACAAAGAAATTTTCTGCATAGAATCGGCATCGAATATCGGGTAAAGTCTTTAAAAGA |
| AAATTTGACTGACAAGAAACAAATAGATCAATTAATTGGATGTTATAAGTTTCTCACCGA |
| TGAAGATAAAATGGGAGAGCGTTTTAAATTTTGTGCCATTTTACCAGCAACACTAAAAAA |
| AATTATGGATAAATATCCAGTGGTTGGCTTCATGTAATATTTTTTTGTAAAAAATTATAA |
| AAATGTGATATACTATGTGATAGTTTAATAATATTGATGTGTGATTAAAAATAATATTAT |
| TTGGATATCAAATGTATTTTAATTTTAAGAATATATACAAAAAATAACCATATGCTTTAT |
| TGTTTATAAAATGATAATTTAGCGGAATGGGCAACCATTTGCCCAATGTCCCCTTTCACC |
| ACAAGAGTAACACCTTCCATAACCCCCATAGTATGCGTCAGGGAGACCTATCATTACGTT |
| CTCTGAAAGATCAAAGTCACTATCGTAATCAAATTCGCTATTGCTACTATTATAGTTACT |
| AGTATCGCTACTACTAGATTCTGAAGAATCTTGTATTTCAATAACTGATGAATCTGTATC |
| TGCCCTACTTGAAAGTCTTGTATTATACATCATAACTCTTTGGGATTTATTACCTTTTTG |
| AGATTGCTTGCTATTTTTTTTCTTATACTCTAATTTTTGCCTTTCCTCCTGCATTATTTT |
| TCTTTGATCTCTTAGCTCTTGAGATGCATTTTCTAACATCTACAAATAATAATAAGCTAA |
| TAAAACACAGCAATATATGAAAAATTACTACTTTTTCTATAACATTATCCAGAACTAATG |
| TTCTTGCTGAAGATGTAATTTTTTTTCTACAAATTGGACACTCTGACTTCTTCTTTTTCC |
| ATGTTTCAATGCAGTACTGACAAAAAGTATGTGAACAAGCCAAAGTTACAGGTTTAATAA |
| ATAAATTAGCACAGATAGTGCACAAAAGTTCATCTTCCATTTCTTCAAATTGATTCTGTT |
| TGAATGTAGAGGAATGTGATACTTGTGGTTGAGAGTTTTGATTTGAACACTGGGAATTAA |
| CCTGATCTTTCTTAGAATTGGTATTTTCGTTGTCACTATTTATCAATGATTCTATATTAT |
| ATTAAAAAATATAGTTAATACAATTATTAACTCAAATTGAAATTACCTGTAGTTATATTC |
| TCCACATTAGCAGGATTTGGTAATGATATTATTGATTTAATTCCAATTTCAGCATTTGAA |
| GTCTCTTTTGAAGAAGTACTTGGTATTTCATGACTAAATAACTGATTCTTATTTCTATTC |
| TTACCTTGTACATAGCTGATATCATTTTTATCCCTATTTGGCAACACTTTTAACTTTTTA |
| TTAGGATTATATGGGCTTATTACAGTTTGTTCTACATTTCTTACATCTACATTAGAATCA |
| CCTTTATTTGAAGATCCCATGACATTGGAGAAATATAAATTTGAATCAATATTAGTATTC |
| GCTTCTATATTTGTGACTGATGTGACTTTCGTTTCCATCTCTATTTCAGATTTGTCAATA |
| TTTTTCAACATATTATCTGCTAACTGACTAAGTAAATCATCACTAATGTCATTAATAGAA |
| AAGGTATCATCATTTAATCTTGGTAAATCTTCAAGATATAGAAGAAATTGGTAAGTGTAA |
| TTCAAGGTTCCCAAACAAATTCTATCATTATGCTTCAATACAGGTGTTGTTTGAAAATGA |
| AAATAATTATCATTAACAAAGATGCCATTTGTACTAGAATCTTGTAAAGTCCATTCATCG |
| CCTCTTTTATTAAGAGAACAATGTGTCCTTGAAATTATTTTATCTTCTATAACATAATTT |
| GAAGTTAAACCTCTTCCAATTGTAAAAGGATCATTTGTAATTTCAATGAGATTCCCGGTA |
| GTTAAATCTTTTAAAATTGGAAAAATCATTTTTACTATTTCATTTCCAAATATATTAATA |
| ATCAAACTAAATATTACTGAAAGAAAGATATCCGGACTAAAATAAATAACAATAGGTCCA |
| CTGTTCGACGTTATTGAGAATAGTTCAAACTTCAAACGCCAAAAATTCTGAAGTTTATTC |
| TTCTTCTGCACGTATTTTGTTTTGAAAGAAGATTATTTCCTTAGTTAATACATAAATATT |
| AAATTTGAAATAGAATTTGATGGAATGTACTGTAACTGATTATTTTTAATATTTAAATTT |
| ACTGTATAAACATGACCTTTAAAAAATGTGTTAATCCAGTAGTTTTTAAAGCAGTTCAAA |
| ATGTTGATATGGAAAAGCTTGCTAAATGTTCAGAAGGTAATATTCGACCAGTTTTACCAT |
| GTCTCGTTCGTATGGGATTAATATCACCTCTGGATACATCAAGAGCATGTACCAACATGA |
| AAGTAAATATTTTAACAGTTGTTAGTGGAATGGAATTAGTCAATTCGATTGTCGCTCTTC |
| TGAGTATTGATTTTCATAAATTAGAAATTGAAGTTAAAAAAGAGCAGCAGCTGAGACAAA |
| AGGGAAATAGTTCCCAAAATGATTCAATTTTAATTGGTAACCTTTCAAATACTAGTATGG |
| CTTTGGAATATGAACGTAGTGATATGACAAGAAGACTTAGTATTTTATTAGGGGAATTAT |
| TGTTCATCCAGTCTCAAATACAAGAACTTCCTGAAACTCCTGATGCTGAAACATATATTA |
| AATCATCAGAGCTATTTGATAATGATATATTTGCGGAAGAATTATCAGATATAATATGTA |
| TAGCTCTAGCTGAACTACCAACCACACTAAATATTTCAAATATTGTGGAGACACTTTTAC |
| ATGTGCACAATGGTCCAGAAATTATATGTAGAGTAGTAGCCAATTTTCCCGATTGTTTTC |
| GTGAGGTATGCACTTATTTAATACAAACTGGTGAAAAACAAGAAGAATCTATATCAAGTA |
| CTATTAGATCTACAACTATAGGGCTTCTATGTCAAATGAATCCATCACAAAGTTTATCTG |
| TTAGAAGTAAATGTGTTGAATTATGTAGAATGCCAGCATTAGCGATAGCATTGTCATTAG |
| GGGATATATGTGGAGATAGTGATGGTGATATGGTTGCCTTTATTAGTGGATTATTACTTG |
| GAAATGATCAAACAATTAGAAATTGGATTGCAATGTTTATAAGAACTGGTCAAAAACGTA |
| AAGGTGAAGCTTCAAGCAATGCATTACAACAATTGCGAGAAGAATTACTGAAGAGGCTAC |
| AAAAAATAATCGATTTTTCTCCAGAAGGTCAAATACCAGATAGTTTAGTAGTACAAGCTT |
| CAGCTCTATTAAGATTATACTGTGCTCTTAGAGGGATAGCTGCTATAAAGTTTCAAGATG |
| AAGAAGTCAATTTGTTGGTTCAGCTATTAACCTCTCACCCTAACCCCACTCCTGCAGGAG |
| TTAGATTCGTATCAATTGGTTTGTGCATGTTGATAGCTTGCCCATCTTTAATTTCACAAC |
| CTGAACATGAAAGACGAAGTATTGAATGGGTTCAATGGCTGGTTAAAGAGGAGGCATATT |
| TTGAATCAGCAAGTGGAGTCACTGCTTCTTTTGGAGAAATGTTGCTTTTAATGGCTATAC |
| ATTTTCATAGTCAACAACTTAGTGCCATTTGTGAACTGACATGTGCCACACTTGGAATGA |
| AAATTGCCATAAGACATAACAATATGAACAGAATGAAACAAGTTTTTACACAAGAAATTT |
| TTACAGAACAGGTAGTAACTGCTCATGCTGTCAAGGTCCCAGTGACTCAAGGATTAAGTG |
| CAAATATGACAGGTTTCTTACCTATTCATTGTATCCACCAATTATTAAAATCTCGTGCAT |
| TTGCTAAGCACAATGTTAACATAAAAAACTGGATATACAAGCAAATTTGTAGTAGCATTA |
| GCCCATTACATCCAGTTTTACCTATGCTTGTTGAAGTATATGTAAATAGTATAATGATAC |
| CAAATTCTAAAAATCTCGAACAAGCTAATAAACCTTTAACTGAAAATGAAATTAGAAGAG |
| TATTCCAAAGCTCTATTTTTGGCCAATATTTTGAAAATAAACAATCTATTTTCACAATGG |
| AATTTGATGTAAATTTTGAAAACCAAGATGTTGTGGTTGACAATACAAGTCTTACTCCTC |
| AATTATTACTGCTATATTATCTTTTGTTATATGAAGATTGTAGACTTAATAATGCCCATT |
| TATTGGCATCTAGTGGTAAAAAAATAAAACAATATACCTCAGAATTTATGTCAGAGCTAC |
| CAATTAAATACTTGCTGCATCATGCTCAAAAAGATCAAAGTTCTTATTCAGGATTATTTG |
| GTCCATTGCTTAAACTTCTGGCAACTCACTTTCCTCATTTAACATTAGTTGAAGATTGGC |
| TTGATGATATGTCAATTCAAACTGAAAAAAAACTTGTCCAAATTGATGAATATATGGTTG |
| TAGCAGCTTTTAATGAAATAGAAACTAATCCATCAAAATGTGCAAAGTTGCTTCAAACAA |
| TGCTTAAAATAGAAGCTATTGATATTTGGCCATTTGCAGAAAGTTTTACTCAATTTGCAA |
| GAAATGTGTTAGGTGAAAACATACCTAGATTTGTTCAAGACCTATATAAAGATGTTTGGC |
| TTAGATTGAACACTGTATTACCTCGTCGTTTATGGGCACTAACTATAAAGAATTTGGTAG |
| ATGATTTTTCATCCATAACGAAAATAGATGTAGCTGAGGATCCATTACAGATAATGCGTT |
| GCGATGAACGTGTTTTTCGTTGTGCTCCTGTTTACGCAATTGTTCTTAGAGTTTTACGAG |
| CCAGTTTAGCCTCTTCCCGAAGTCAATTAACACAACATTTACAGTCAAATCCAAAATTAG |
| ACTCTCATGGCCAAATTTTAAATGAACCGGATAGAGAAGAAATGTGTAGAGCTACTACTG |
| CAGCTCAGGAAAGCGCTGCCGTACAAATGCTTTTAGAAACTTGCATAGAAAATTATTTAG |
| ATAAAACCACACCTGGTCGCCAATGGGCATTACAAGAAGTACGTTCATTGGTTTGCTCTT |
| ATTTACATCAAGTTTTTATAGCTGATACTATGCTGTGCAAATTAGTACATTTTCAGAGTT |
| ATCCAAGTGAACTCTTGGAAGTTGTTATCAAAGGAGTGCCATCTATGCATATATGTTTGG |
| ATTTCCTTCAAGAGCTAATGCAACAACCCAGTTTAACCAAGCAAATTTTTGCTGTTCAAC |
| TGTTATCACATTTATGTGTTCAATATGCTTTGCCAAAAAGTCTAAATCTCTGTGTAACAG |
| CACTTAATCTACTATATGCACTTCTTGGAGGTATTTCAAGCGTTCAACGAGTTAAATTGT |
| TCAAGCCTGTATTGCCAGCGTTGGTTAGAATAAGCGAAGCTTTTCCTCCCTTAACTGATG |
| ATATTGTAAATTTATTAATGCAGTTAGCCAGAATATGTGAAAGTCAGGCTTCTTTAGCTA |
| GTCATTTCGATAGCCAAAGAGGAAAAGGGTTGGAAATATCTGCACAAGAAAGTGCAGAAT |
| TATGTGATTTAACGAAAAAAACATTTACAGAAATTCTGGATAAAACTGTATTACGAACGA |
| ATGTCTATAGACAAGAATAATTACACGGTAATATATTTTTAAGAATATATGATTCTAATT |
| TGTTTCTGATCGTTTTAACCCTACCACCACTTTTTAACTTTTTTATCTTCAATAATTGAT |
| TGTTTAGCCTAAGCGCTTACTGGAAAACCGACGCGGTATTTGGCAAATCGCAATTGGCAA |
| GCAAGCTGCACACACATGTTTTATAGTTGCCCAAAATTACTCGGTGCGGGAACAACGAAT |
| ATTTGAGAATGCATGTGTACAGTGATTCATTTTCAAAAAGTCTAAAAATTCCAGCATGTT |
| TCCGTTTCACCACTCTATTGTATAGACGAAATAAACTAAAACATTTGACGGGCCCCAAAC |
| TTGTGCAAATATAATCTATAAGTGCAAACGCAAAAATTTGTCTTTAACTCGACTCGCAAT |
| ACCAATACAAGCAATGGGAAAACACCAAATGGCAACCTATACGCACAGTGCGACTTGCCG |
| ACTAGCGAATATTTGTTATTTGAGCCTCACCAAATACCGCATGTGCAAAAAGGCAATTGA |
| AATGTACAAAAATAATATGATTTAAAAACATAAAGTAACAAATATTTCGAAAATGCAATT |
| TAATATAAATTAGAAAATTAATATATAATAAAAACAGATTTTATTGATATAAATATTCTG |
| CTCTGAAAAGTGACTGACCTTTATATATATTATATAAATTGTAAAATATGTAATAAAACA |
| AAATAAATATTTCTACAATATTCCATACTACAAATTTAAGTACAAAACAATGATGTGCAA |
| TATTATAAGTCATGATACACAGTCCTGCATTTTTTAGCATCTGTTACTATAGAAAACATT |
| ATTTTGTATATTTAAACAAATCAACTAACACTGCCTGTTCTTGGCGTTTAATATTTTTCA |
| TATCAAGTATCTTATGAAATTCTGTAAGCTGACAATCTGGTATCAACTTTTTGCATTGAT |
| CTATGAAATTTTTTTGAGGTTCTATAGGCGTCATCACTACTTTAAGGATCATTTCAGCTT |
| TAGTCATCCCTTTTGTTACTACTTTTGAATAAGCGGTTGGTGCTGGTCTATTAATTTGAG |
| AGGATATTGAAGGTAGATTTAATAGAACTGTTTTAAGCATATGTGTATCAAGCAGAAGCT |
| GTTCTGCACCAACTGTATTAATAGGCTTGCACTTATATATACTTTGTATAAATTTTGGTA |
| TGAAGCTATTTGCGAATTTTATACAAAATTGTGTAAAATACTTTCTTGAATGGGCCAGAT |
| TGTCCCTAATAATAGGAATGGTTGTCTTTAAATGTGTTGTTATAGAAGTAATGTAAGGAC |
| TTTGATCACCAACAGCATCAATATTTTGCCAAGGAATCTTTGACATAGCTGTTAGAGATG |
| GCTCACATGCATTTTCAAGATCCTGTACTAACAACTGAATACAATTAGAAATTACTTTGT |
| GAAATTGATCCTGTTCTTTTGATAAGTCTATTTGGTCCGCTAATAATGGATCAATTTTCT |
| CCTTTAGTTTATCTTGCAACTGCTGTGTTGTTTCTAAACAATATTCGGCAGTAGTTAAAA |
| TACAACATATTTTGGTTTGTTCATCTTTTGTAAACCTAATTACTTCGCCTTCTTTTAATA |
| AAGATGAAAAATTTTGAATTAAACCTGAAGTTGACATTTTTTGCAAATCTTTAGTAAAAT |
| TTTGTACTGAACTACCTAAAGATTGCCCTTCTATTTTTGGTAAATTATTATAAAGTAATT |
| TTTCAGAATATTCCTGAAGATATTTTTGAAATATTCTTGTTAGACTGAGCATTGACTGTC |
| CTTTATCCAGTTGTGTGCATTGTATCATGCTTTTTTTATAAAATACAAATAAATCAGGAC |
| AAGAAGATAAAATTGCTGCTTGTGTCTCTGCATTTTCAATTGGTCTATGTTGTTTTTCAT |
| CTTGTACAAATCTGTCAATCAAATCAGCTAAATTTCTATCCAAGCTCTCTATATAAATTT |
| CCAAGTGAGTAATGAAACACTGACCAATTAATCCATTAAATAGAGATTCATTAAAATCTA |
| ACTGCGGATTAGATACATTTTTTTCACCTTTTTTGTTTTCTGCCTTCAATTTTTCATTAG |
| AATCCAAGTTTTCTCTAAGAGTTATCCCAGTGAATCGTTTAAATAGTAAATTTTCAAAGG |
| CAGATGTTTTTTGAATGGCATATAGTAGTAATTTAACATCAATTTCACCTTTTCTTTTTG |
| ACATTATTTTTGCTAGTTCTTCACGAGTATTATGACAAAACTGAACTACAATTCTTTCAG |
| AAACTTCCCAATTTTGTGGAAACATATTACCCATTCTATCCTCAAATTCTAAAAGATGTC |
| TTTTTATCCAAGCATATCTTTTGTCAATTTTATCTAACCATGCCGTATCTTCAGTTTCTT |
| GAAACAAATGGTTATATTCTTGTAATTGCAACCCCACAAACCATTTTAGTAACTCTCTTT |
| TAACTTTTGGATTTAGTATAGATACAACAAGACATGCTTGTTGTAACTGTTTATTTGGTA |
| CAATATTTTTACTGTTTGTTCCTTCAAAAGCTTCCTTAAAATCATGGGTTATTTGTTCAG |
| CTAACTCAACATGTATGCTTTTGACTTGATCACTTAAATTCTTTATTTGGGGAATATCTG |
| AATAATTTTCAAAATGTGTCATCACTTCACTAATTGCTTGTAAAGGCAAAGCTATTTCTC |
| CATATAAACGTTTTTGTGTTAATGATTTTAAAGTATCAACTCCTCCAACAAGCATATGAA |
| GATGATTTAATGTGGTTATGGCCAAAGTTAAATTTCTTTTAGCACAATCTAATTGTTTAA |
| TATCTCTTGTAATTTCTCTAACCATTTCTTCTGACTTTTCTGCTCTTTCTTTAATGTCTT |
| TAATATGTAAAAATAACTGTTTTATAATTTTTTGTGCCTCATCTAATGCCTCCCTTCCAT |
| CAGAGCTAGCCTCGATCTGACTTCTGATGACTGTTGAGATTTCATTATCAATAGTGCTAA |
| TTTGATTTTCCATTTTAATAACCATTTCATCGATATTTGATAAAGATTGTTCTGTTGGAA |
| ATAAAGAATTTATGTAGTCAATACTGTTAAAATCAGGTTCATCCAAGGAATCTCTATTAG |
| GCAACACCTCATCTATAGCTTTTTGAACTTCTGGAGGAAAATTAATAAAAAATTCAGAAT |
| TATCCTCATCTAATAACTCATCCTCAAATTCCATGATGTTCAATTTGTTTTCAATATGTA |
| ATTGGCGCTTTTGCACAATATAAAGCAATGAACACAAAATATGAATTGAATTCTTCTATT |
| CTATTTATCACAGATAATGGCAACTTTATACATTTCACATATAGTATAGTGGTTTATTAT |
| TTTAAAACATTTTTTTACATGTCAAAATTAAATGTAAATATAAAGCATATGTAATCCTGC |
| AAGAAAAATAAACAAGAATGACAGATGCTATCAGTGTAGCCATATTGCACCGTAT |

| >*Cbuq74007_g1* | |  |  |
| --- | --- | --- | --- |
| TTTTTTTTTTTTTTTTTTTTTCGAGCTTTCATACTATCTTTATTGTTTAGTTTATTATAA | | | |
| AACAAGAACAATTAATATTTATGATAAAGCGTTAGAATTATATTAATGTTTATGGCCAAA | | | |
| ATGTCTTCCATAACAAACAAGCAAATTATGGGCTGTGTCCTTCTCATCGTCGTGATCGAC | | | |
| CAAACACTCGCTATAAATCTTATCCGCCTGTTCCTTATCGGTAATGAAACTGGATATTTT | | | |
| CTCTTTGACTGATGTTTTGTCTATTTTTCCGTCGGGGTGTTGCCAGCCAAGCGTTTTCGA | | | |
| CACACAAAGCGCGTGAGCTTTTAAGCTAGGTCCTACTACTTTGGAGCTTTTGTATGCTTT | | | |
| TTTTTCTTCGGGATCAAGTTTGGTAGCCGCGTCACCATTACATTTCTCATGGGCTTTCTT | | | |
| GACCTTTTCTTTGCTTTCTTCGACTGTGAGAGCCAGAACTGTTGACAAAATAAAGCATAA | | | |
| AAGAATTGCGATAGACCACTTCATATTGACGGTGATTTTTTATGCTTCTTCAATTTAATG | | | |
| AAATTAACTACATAGTATTCAAGTGGAGCTTTTTATACTGGTGTGCAATGTGTTGTGAAG | | | |
| ACCGTAAATGATAAATCTAAAAGGTCAGCTTTTAATAAAGCATCTCTCTAAAGGTCAGAA | | | |
| ACGCAAACAAATGGATTTAGCACCTTATCA |  |  |  |

| >*Cbuq74056_g1* |  |  |  |
| --- | --- | --- | --- |
| GGGGGGGACCATCTCTAAGGTTACCGGTGTTACAGTGATAAAATGAAGTATTTTATTTTA | | | |
| ATTTCTGTTCTTGTTAGCGTATTTACATGTGGCTTTGCTGCATCGAGAGCTACGTGGACT | | | |
| CAAAAATTCTTTAGTTTTACAAACGAATGTATAGCTGATACCGGTATAGAGGCAGACATT | | | |
| GTCCAAAAAGCTTTGCAAGGCCACATTACCAATGATCCTAAGTTGAAGACTTTCCTATTT | | | |
| TGTATGACGAAGAAAGGCGCACTACAAAACGCGAACGGCGAGGTCCAGATTGAAGAGTTC | | | |
| AAAAAACAACTGCCCAGTCTTGTCGAAAATCCTGAAACTACTATTGAGTTGGTTAGGAAA | | | |
| TGTGTTTGGAAAGAGGGAACCCCTGAAGATATTGCATTGCAAATATATGGATGTTTTTAC | | | |
| AAAACCGATTCTAATAAATAATAGATTAATATGATGACATTTTAACTAAATGTTCTTTTA | | | |
| AAGTTTTAATAAAGTATTGGGCCTATAAAAAAAAAAAAAAAAAAAA | | |  |

| >*Cbuq7577_g1* |  |  |  |
| --- | --- | --- | --- |
| AGATGGAAATTCGGTGACCTTCGACTGACTCTGCATTTTTAAGTATAAAATGGCCTGGGT | | | |
| CTGTGAGAAAATCCATCAATTGACTCACAATCGTTCATTCAACAGTGTTGTTACATAGCA | | | |
| AGTGTCCTGCATTGCAACATGAAGCTTTTACTGGTCTTGGCTTTGGCGCTGGTAGCTGTC | | | |
| AATGGGCTTAGCGAAAGCTTAGTTGATGAGATGAAAGAGAAGTTACAAAAATATGGATTG | | | |
| GAATGTGCTGAAAAAGAGAAAGCATCAGAAGAGGATATCCAAGCTCTGATGAACCACGAA | | | |
| CGACCAGTCACCCATGCCGGAAAATGCACTATTTTCTGTACATTCAAAAAATTCGATTTG | | | |
| ATGAAAGAAGATGGATCCTTTGGTCCCGGTGACATGGACTGGATTGAAAGAGCCAAAGCT | | | |
| GACGATGCGGAATTCATGGAAAAACTAACGGGTATACAATCCACATGTGAAAAGACAGTT | | | |
| CAAATAGACTCTGACCCGTGTGAGACCGCATTACGAGCTGCAAAATGTGCTAAAGATGAA | | | |
| GGCGAAAAACTTGGAATTACCAGTTTTTAATTTGTTTATCATAAAAAGACCTTGTAATGT | | | |
| TAAATATACTGAATAATAAACTAAACTGCATTTAAATTAATGTTTTCATTTTTAAAT | | | |

| >*Cbuq85742_g1* | | |  |  |
| --- | --- | --- | --- | --- |
| AAAATAATCGTTTTGATTCGGCATATAATAGTGGTGCAATATTTTTCCTTGTTTATTATA | | | | |
| GAACTATTCATTGATTCCGTGAAAAAAAATCAGCGTTATATATGAAAATAATAAATTATC | | | | |
| ATTGGGTCGCCGACGAGTTCTTCACGCATCTCTTAACGCCATCACCATAAGGCTGATAAT | | | | |
| ATCCCCGTAAGACTGGCCTCACTCTTGATCCGTGATAAACTTTGTCTTCGCGATAAAGCG | | | | |
| TTTTACTTAACTCAGTATCCATATACTGCGCTACAAACTTGAAGAAAAAGTATTGAAGAA | | | | |
| CGTTTATTTTTACTTATTTTTTGGTCTCTCTTGTTTTGGTTGTTTCTCCGGCTGCGTAAA | | | | |
| AATGAAGTTTGTTGTTCTGTTCTGTGTCGTTTTGTTATTGGCAACGGTTGTATCAAGTAA | | | | |
| AAAGCATCACAAAAACAACAACGAGGTAACACCAAAAAAAGCTTTTAAGGAGTGTCAAAA | | | | |
| AAATGCAACCACCCGTATAGACAAACAAGCTGTTAAGAAATATAAGAAGAAGGAAGTAGA | | | | |
| CAGTATGCCACAAAATTACGGCGAGCATCTTCTTTGCATTTATAAAGCAACTGGATATAT | | | | |
| TGGAGAAGATGGTGTCGTAAATCAAGACGTTTTGAAGAAGAAAATAACGAAAAAAGCCCA | | | | |
| ACCAAGTCAAAACGTTGATACCTTGCTACAGGAATGTGGTGCTGCGAAGGCAGATCCCAA | | | | |
| ACAGACAGCTATTAACCTCGACTCGTGCTTAACAAAGAACAATCTCTAAAGATGAACGAA | | | | |
| AAATAAACGTTCTTCCATTATCTCGGTTTTAGGTTATGTATCTTTTAAGTCGTTAGGCAA | | | | |
| CCAGAAACCAACTATCATTCAATACGTATCGTGCCAAATGTATTTATAAGTTTCAGTTGT | | | | |
| ATAGTTTTGTAGTATTTAATATACATTAATAAAATTTAAGACAATAAAATTGTTTATACG | | | | |
| AAAAATAAACGTTCTTCCAT |  |  |  |  |

| >*Cbuq97376_g1* |  |  |
| --- | --- | --- |
| TGCCACTGTAGTTGAACAGTTAAATCGCATACGGTGTTTACGAGTTAGTCGAGTCCTGTC | | |
| CCAGACCAAGCACTCGGCAATTTACCGACCATATATAAACGGTTTTGGACCGTTAATCTT | | |
| CAGTGCTAATACCTTTTAGCCATCATCATGAGTGGTCTACTTGCTTTGTGTGCGATCTTA | | |
| GCCACGGCTTTGGCCATTACGGTAGCGTATGACTTCGAAGACGCAGATTTCAATCAGTTT | | |
| TTAGCGGATGACCTCGAAGACGGGTTAGATACGTTGGATTCTGCGTTTGTTCACTATAGG | | |
| GTCAGGCGCGCCGAAGATGCCAACCCACCGGCTCAGTCCGGCGATGATAAATGTAAGAAG | | |
| AGACGTAGGAAACCATCGTTGTGTTGTGCGGATGATATTATCGACCAGCAACATGAGAAA | | |
| GATCGCGAAACCTTCAGATCTTGCTTCAGGGAAGTGTTGGGTGTTGAGAAGTCCGGTCAT | | |
| CATCGTAGAGGTGACCCATTTAGTTGTAAGGAGGCTGAAAAACGACGTAATGACATGACC | | |
| TGTGTTACTACATGCTATGGTCAAAAGAAGGGCTTCCTGGATGATCAAGGAAACCCCATA | | |
| CCAGAAGCTCTAACCAAGAGTCTGAAGGACGCCTTCGCTAAAGAAAGTTGGTTCGATGGT | | |
| GTAGCGGATAAAATTGTGACCACCTGTCTTAAGGAGGCTGATAATGCCACACAATACCAA | | |
| CCTAAACCGTCTTCCGATGACATCAAACTATGTAATCCATCAGGTCTCACTCTGAAACAC | | |
| TGCCTGTTCAAGCAGATCCAACTCAGCTGTCCTGCTGACCAGATTAAAGACCAAAAGGCC | | |
| TGCGACAAGTTTCAAGACAGAATCAAGAAAGGTTTGGATGATGTGGAACCACAACCCCCT | | |
| CCGCCATTTGATGGTCCCAGAGACGACTGAACAGGTCGAGTTAAGCTGATTCAGTTGAAT | | |
| CGAGTATGAATTAGCGAGTTGGTATATAAGATAAGATACTTAATTTATATTTAAAATATT | | |
| ATAATAAATTTTATACAAAATTATTATAAAAATAAATAAAAACCCCAAA | |  |

| >*Cbuq97535_g1* | |  |  |
| --- | --- | --- | --- |
| TTATTTATTTTTTTTTTTTTTTTAATTTTGTGAATTTTATTTTAAATTTAATATAATATA | | | |
| TTTGTTAATAATCTAGAATATAGAGGCGTGAGACGGAGTTTTCTCATAATAACAAACGAA | | | |
| CGTTTGGTACGCCGTTTCTTCGGGAGAACCTTTGTTCTGGACGCAAGCAGAAATCAAACT | | | |
| GTTGACGGTTTCGGCGTTATCGACGACGCTAGACAATTTCTCCTTCAAAACTTCATGTTG | | | |
| AACATCACCACTGGCGTTTTGCAAACCAATTTTCTTCGAGAGGCAGAAGGCGAAAGCTTT | | | |
| TAACTTGGGATCATCGCTGAAACTCCCTTGTCTAGCTTTAAGGACGAGTTCCTTGTCCAC | | | |
| GCCAGTTTCAGCAATACACTCCTTTCCATAGTTGAGGACCTTTTGCTTCTGTTCATCGCT | | | |
| CAAATCAGCCAAAACATAGATGAACACGCACAAAATAGTTGAGATAACCAGTAATGATTT | | | |
| CATCTTGCTTCTATTTTGTTTATTATTTAATTC |  |  |  |
