# Supplementary material for: The developmental transcriptome of the bamboo snout beetle Cyrtotrachelus buqueti and insights into candidate pheromone-binding proteins
Source: PLoS One. 2017 Jun 29;12(6):e0179807. doi: 10.1371/journal.pone.0179807 (PMC5491049; doi:10.1371/journal.pone.0179807)
Supplement: S3 Table — (DOCX) [file pone.0179807.s035.docx]

| Gene |  | 5'--3' | Tm | Product |
| --- | --- | --- | --- | --- |
| *GAPDH* | F | CAGCGTCTTCGGTGTAA |  |  |
|  | R | GACCCAACTCAAATCCC |  |  |
| *Cbuq12614_g1* | F | ATTTTACATTTCTCCACGATTGC | 58.3 | 100 |
|  | R | TGAATGACGAGGGCGAAGTA | 59 |  |
| *Cbuq16395_g1* | F | TGGGTTGGACTGGCATTTG | 59.6 | 98 |
|  | R | CCGCTTTATTGGCTTCACCT | 59.6 |  |
| *Cbuq25979_g1* | F | CGAAATCACAGCAAATCGTCAG | 60.9 | 153 |
|  | R | CCTCAAAAGCGTAAGGAGCATC | 61 |  |
| *Cbuq29237_g1* | F | TGTTGGTGGTGTTTCATACGAG | 58.4 | 148 |
|  | R | TTTTGGCAGCAGTGAAGACAG | 58.9 |  |
| *Cbuq37516_g1* | F | CTCTTTGACCGATGTCCACG | 58.5 | 165 |
|  | R | TCAACAAAGAAGTGACCGCTAAG | 59.3 |  |
| *Cbuq74007_g1* | F | AAATCTTATCCGCCTGTTCCTT | 59.3 | 85 |
|  | R | CACCCCGACGGAAAAATAGA | 60 |  |
| *Cbuq74056_g1* | F | AACGGCGAGGTCCAGATTG | 60.6 | 96 |
|  | R | CCAAACACATTTCCTAACCAACTC | 59.9 |  |
| *Cbuq67219_g1* | F | ACCTCTCACCCTAACCCCACT | 59.1 | 135 |
|  | R | AACCAGCCATTGAACCCATT | 59.2 |  |
| *Cbuq7577_g1* | F | CGAACGACCAGTCACCCAT | 58.4 | 207 |
|  | R | CACACGGGTCAGAGTCTATTTG | 59.4 |  |
| *Cbuq85742_g1* | F | GAATGTGGTGCTGCGAAGG | 59.5 | 158 |
|  | R | GGTTTCTGGTTGCCTAACGACT | 60.2 |  |
| *Cbuq97376_g1* | F | CGTTGGATTCTGCGTTTGTT | 58.6 | 195 |
|  | R | CAACACCCAACACTTCCCTGA | 60.5 |  |
| *Cbuq97535_g1* | F | AGAGGCAGAAGGCGAAAGC | 59.6 | 87 |
|  | R | CTGGCGTGGACAAGGAACT | 58 |  |
